# Folate can promote the methionine-dependent reprogramming of glioblastoma cells towards pluripotency

**DOI:** 10.1038/s41419-019-1836-2

**Published:** 2019-08-08

**Authors:** Racha Zgheib, Shyue-Fang Battaglia-Hsu, Sébastien Hergalant, Maelle Quéré, Jean-Marc Alberto, Céline Chéry, Pierre Rouyer, Guillaume Gauchotte, Jean-Louis Guéant, Farès Namour

**Affiliations:** Université de Lorraine Inserm 1256 NGERE, F-54000 Nancy, France

**Keywords:** Cancer metabolism, Cancer stem cells

## Abstract

Methionine dependency of tumor growth, although not well-understood, is detectable by ^11^C-methionine positron emission tomography and may contribute to the aggressivity of glioblastomas (GBM) and meningiomas. Cytosolic folate cycle is required for methionine synthesis. Its dysregulation may influence cell reprogramming towards pluripotency. We evaluated methionine-dependent growth of monolayer (ML) cells and stem cell-like tumor spheres (TS) derived from 4 GBM (U251, U87, LN299, T98G) and 1 meningioma (IOMM-LEE) cell lines. Our data showed that for all cell lines studied, exogenous methionine is required for TS formation but not for ML cells proliferation. Furthermore, for GBM cell lines, regardless of the addition of folate cycle substrates (folic acid and formate), the level of 3 folate isoforms, 5-methytetrahydrofolate, 5,10-methenyltetrahydrofolate, and 10-formyltetrahydrofolate, were all downregulated in TS relative to ML cells. Unlike GBM cell lines, in IOMM-LEE cells, 5-methyltetrahydrofolate was actually more elevated in TS than ML, and only 5,10-methenyltetrahydrofolate and 10-formyltetrahydrofolate were downregulated. The functional significance of this variation in folate cycle repression was revealed by the finding that Folic Acid and 5-methyltetrahydrofolate promote the growth of U251 TS but not IOMM-LEE TS. Transcriptome-wide sequencing of U251 cells revealed that *DHFR*, *SHMT1*, and *MTHFD1* were downregulated in TS vs ML, in concordance with the low activity cytosolic folate cycle observed in U251 TS. In conclusion, we found that a repressed cytosolic folate cycle underlies the methionine dependency of GBM and meningioma cell lines and that 5-methyltetrahydrofolate is a key metabolic switch for glioblastoma TS formation. The finding that folic acid facilitates TS formation, although requiring further validation in diseased human tissues, incites to investigate whether excessive folate intake could promote cancer stem cells formation in GBM patients.

## Introduction

Methionine dependency is a common feature of most cancer cells^1^. It is characterized by the cells’ inability to grow without methionine even in the presence of its precursor homocysteine^2^. Glioblastoma (GBM) is one of the most aggressive brain tumors and current therapeutic strategies remain ineffective in delaying its progression^3^. GBM is composed of heterogeneous tumor tissues^4^, and is enriched with self-renewing stem-like cells^5–7^. Meningioma is the most frequent non-glial primary brain tumor and accounts for 30% of brain tumors^8^. Methionine dependency might flag the aggressive grade of these brain tumors and explain their increased uptake in positron emission tomography of ^11^C-methionine^9–11^.

**Fig. 1:**
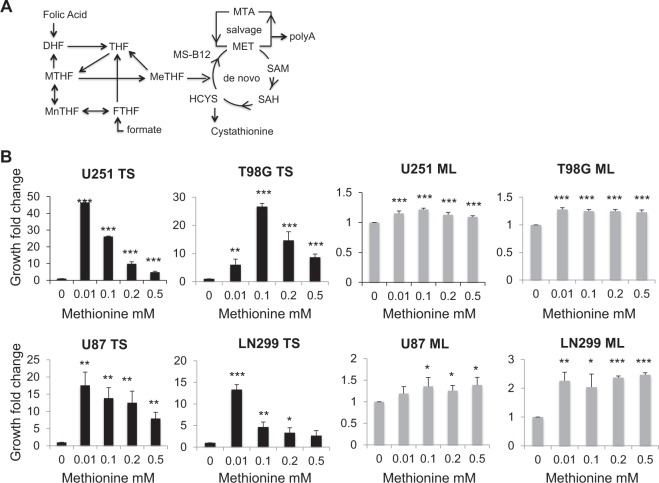
Methionine increases the growth of tumor spheres derived from U251, T98G, U87, and LN299 glioblastoma cell lines. **a** Methionine synthesis de novo and salvage pathway; (**b**) methionine, up to 0.01 mM for U251, U87, and LN299 and 0.1 mM for T98G cell lines increases TS growth. Cells are cultured in methionine free media than methionine is added to reach the indicated concentrations. Samples are compared to cells grown with no exogenous methionine addition, n = 9; *p < 0.05, **p < 0.01,***p < 0.001; DHF: dihydrofolate, THF: tetrahydrofolate, MeTHF: 5-methyltetrahydrofolate, MTHF: 5,10-methylenetetrahydrofolate, Mn: 5,10-methenyltetrahydrofolate, FTHF: 10-formyltetrahydrofolate, HCYS: homocysteine, SAH: S-adenosylhomocysteine, MET: methionine, SAM: S-adenosylmethionine, MS: mehtionine synthase, B12: vitamine B12, polyA: polyamines, MTA: methylthioadenosine, ML: monolayer cells, TS: tumor spheres

Under normal conditions, homocysteine can be remethylated to produce de novo methionine required for methionine-dependent processes such as the production of SAM (S-adenosylmethionine). This remethylation is catalyzed in the cytosol by methionine synthase, which transfers the methyl group of 5-methyltetrahydrofolate to homocysteine in the presence of vitamin B12. Cytosolic folate cycle is mostly fueled by the one-carbon units exported from mitochondrial folate cycle^12^. In addition to homocysteine remethylation, methionine can also be regenerated from methylthioadenosine, a by-product of polyamines synthesis via the salvage pathway (Fig. 1a). Experimental evidence suggested that mutations in enzymes of either the de novo or salvage pathway of methionine can be the cause of methionine dependency^1^. Increased cellular needs for methionine-requiring processes can also cause methionine dependency. Indeed, human pluripotent embryonic stem cells (hES) require high amounts of methionine and express high levels of enzymes involved in methionine metabolism^13,14^. Methionine deprivation in these cells eradicates undifferentiated hES cells and facilitates their differentiation. Just like embryonic stem cells, stem-like cancer cells derived from breast cancer cell lines are much more methionine dependent than their differentiated counterparts (adherent monolayer cells)^15^. Stem-like cancer cells and normal pluripotent stem cells thus may share common rewired methionine metabolism.

We hypothesized that the dysregulation of methionine metabolism could influence cell reprogramming towards pluripotency in GBM and aggressive meningioma. To investigate this, we studied the capacity of GBM cell lines U251, U87, LN299, and T98G, and WHOIII meningioma IOMM-LEE cells to produce stem-like cancer cells grown in vitro as tumor spheres (TS)^16^, according to methionine availability. We compared TS formed under non-adherent culture condition with their non-stem-like counterparts (obtained as adherent monolayer cells produced under adherent culture condition) using transcriptome-wide sequencing, methylome, and targeted metabolic profiling. Our data here indicate that relative to their differentiated counterparts, the methionine dependency of GBM TS is, in part, a consequence of reduced availability of cytosolic folate isoforms, triggered by a repressed cytosolic folate cycle. In particular, the reduction of 5-methyltetrahydrofolate in GBM TS rendered their growth rescuable by either folic acid or 5-methyltetrahydrofolate in the absence of methionine. Transcriptomic data further implicated the involvement of upregulated mitochondria cycle accompanying the repression of cytosolic folate cycle in the reprogrammation of U251 TS cells.

## Results

### Methionine is required for the growth of tumor spheres but not monolayer cells derived from U251, T98G, U87, and LN299 glioblastoma cell lines

Differentiated monolayer cells (ML) and tumor spheres (TS) could be derived from 4 glioblastoma (GBM) cell lines U251, T98G, U87, and LN299 cultured in a methionine free-medium to which exogenous methionine was added in incremental amounts up to 0.5 mM (Fig. 1b). The presence of methionine was obligatory for GBM TS growth while only moderate dependency on methionine was detected for GBM ML. Most importantly, optimal methionine concentration in the culture medium was required for maximum TS growth and was found to be 0.01 mM for U251, U87, and T98G, and 0.1 mM for LN299 cell lines. Taken together, these results show that methionine is essential for GBM TS formation and that above the optimal concentration, methionine inhibits further TS formation.

### U251 tumor spheres exhibit stemness and folate cycle signatures

**Fig. 2:**
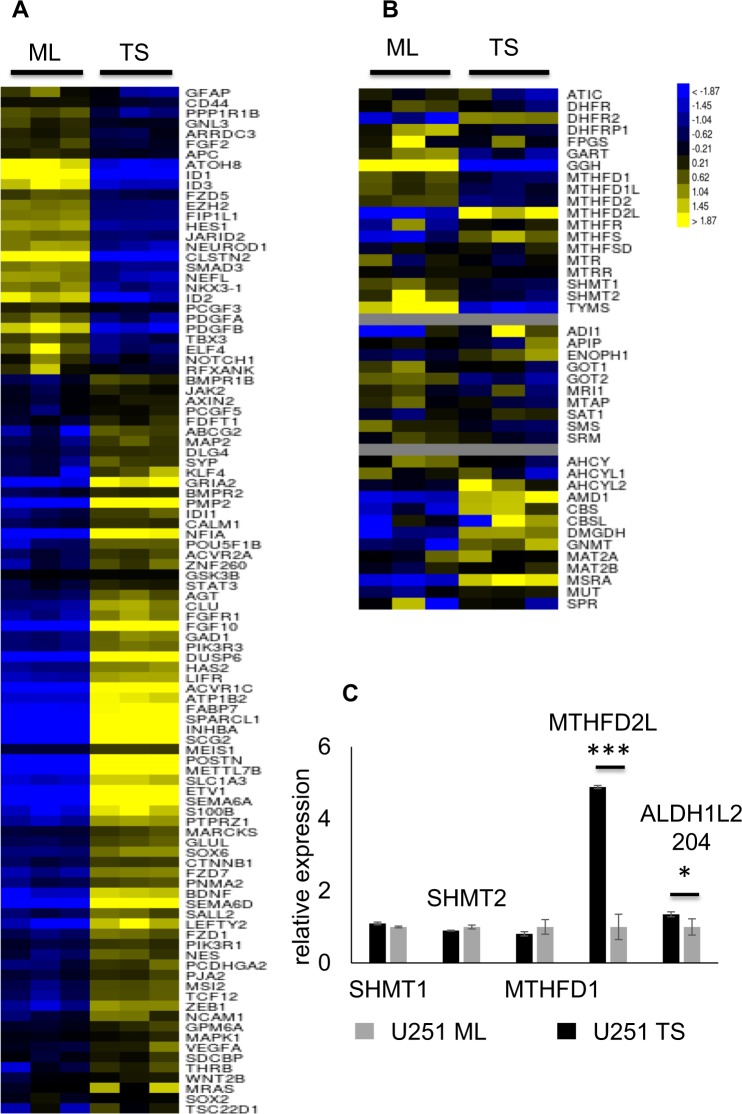
U251 tumor spheres exhibit stemness and folate cycle signatures. **a** Hierarchical clustering of 100 genes involved in neural stemness reveals that 72 genes are over-expressed (yellow) and 28 genes are under-expressed (blue) in TS, *n* = 3; **b** folate signature is characterized by reduced transcription of cytosolic folate cycle genes in TS, including SHMT1, *MTHFD1*, and *DHFR*, and elevated transcription of *MTHFD2L* implicated in mitochondrial folate cycle, *n* = 3. **c** RT-qPCR data, *n* = 3, confirm the elevated expression of mitochondrial folate cycle enzyme *MTHFD2L* and ALDH1L2 204, an alternative transcript of *ALDH1L2*, in TS comparatively to ML cells; However, the expressions of *SHMT1, MTHFD1, and SHMT2* do not vary significantly. *p < 0.05, **p < 0.01, ***p < 0.001, TS = tumor spheres, ML = monolayer cells,

**Fig. 3:**
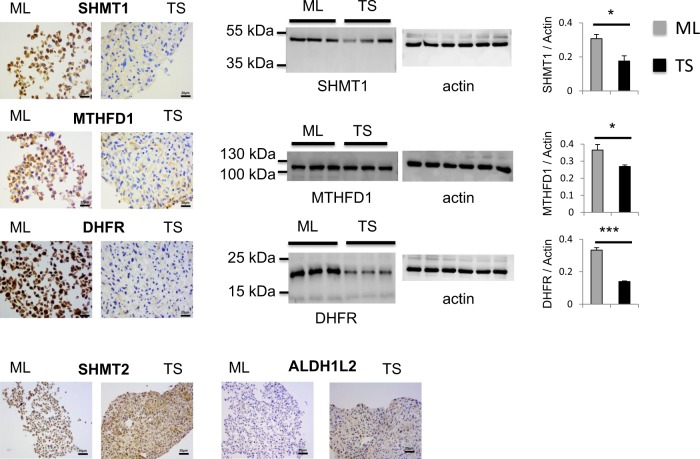
Folate cycle is reprogrammed in U251 tumor spheres. SHMT1, MTHFD1 and DHFR are repressed in TS as they display stronger signals by immunohistochemistry and western blot in ML compared to TS, n = 3. For Western Blots, the same gel was stripped and blotted with actin antibody; SHMT2 and ALDH1L2 are upregulated in TS vs ML as shown by immunocytochemistry, n = 3. Scale bar = 20 μm. ML = monolayer cells, TS = tumor spheres

A total of 23,048 genes were analyzed via k-means clustering after transcriptome sequencing of the U251 glioblastoma (GBM) tumor spheres (TS) vs monolayer cells (ML) (*n* = 3). This led to the identification of 2 large clusters, one with 4515 under-expressed and the other 4756 over-expressed genes. These clusters unveiled a drastic transcription prolife change (40.2%) associated with the reprogrammation of U251 ML to TS (Fig SI 1). RNA sequencing data with at least 1.5 fold-altered genes is displayed in Table SI 1. Our interest focused on neural stemness (Fig. 2a) and folate metabolism (Fig. 2b) signatures. The stemness signature was derived from a consensus set of 100 genes. Among these, 72 were upregulated in U251 TS and included *PMP2* (+352 fold), *SEMA6D* (+64 fold), *SEMA6A* (+58 fold), ACVR*1C* (+58 fold), *SCG2* (+201 fold), *POSTN* (+863 fold), *DUSP6* (+40 folds), and *METTL7B* (+18 fold), and 28 were downregulated and included *ID1* (−50 fold), *ID2* (−10 fold), *ID3* (−10 fold), *ATOH8* (−16 fold), and *CLSTN2* (−54 fold). One-carbon gene heatmap revealed that transcription of genes involved in methionine synthesis did not vary. On the contrary, transcription of genes involved in the cytosolic folate cycle, like *DHFR*, *SHMT1*, and *MTHFD1*, was downregulated and transcription of MTHFD2L involved in the mitochondrial folate cycle was upregulated. Heatmaps represent the average gene expression level and don’t uncover subtle variations in same-gene transcripts. Careful analysis of transcripts expression revealed that certain transcripts of SHMT2 and ALDH1L2, also implicated in the mitochondrial folate cycle, were elevated suggesting that mitochondrial folate cycle might be activated in U251 TS (Table SI 1). We verified the transcriptomic data by analyzing transcript and protein expressions in U251 cells. RT-qPCR results showed that in TS vs ML, while *MTHFD2L*, and an alternative *ALDH1L2* transcript, *ALDH1L2 204*, were elevated (Fig. 2c), *SHMT1, MTHFD1*, and *SHMT2* remained unchanged. Using either immunocytochemistry, Western blot, or both, we confirmed the lower protein expression of SHMT1, MTHFD1, and DHFR, in U251 TS, and the higher protein expression of SHMT2, and ALDH1L2 (Fig. 3).

Furthermore, almost all relevant genes involved in cell cycle were downregulated in U251 TS, including genes coding for cyclins (A, B, D, and E), cyclin kinases (*CDK1*, *CDK2*, *CDK4*), M-phase inducer phosphatases (*CDC25A, CDC25C*), multi-interacting processivity factor *PCNA*, and checkpoint kinases (*CHEK1*, *CHEK2*) (Fig. SI 2). In addition, two large genomic histone clusters HIST1 (6p21-6p22, over 600 kb) and HIST2 (1q21, over 150 kb) were nearly entirely silenced in U251 TS (Fig. SI 3).

Collectively, these data confirm the stemness and quiescence feature of the U251 TS and unveil a potential folate cycle reprogrammation associated with the transformation of differentiated GBM cells into stem-like GBM cells.

### Folic acid promotes stemness of U251 glioblastoma tumor spheres

**Fig. 4:**
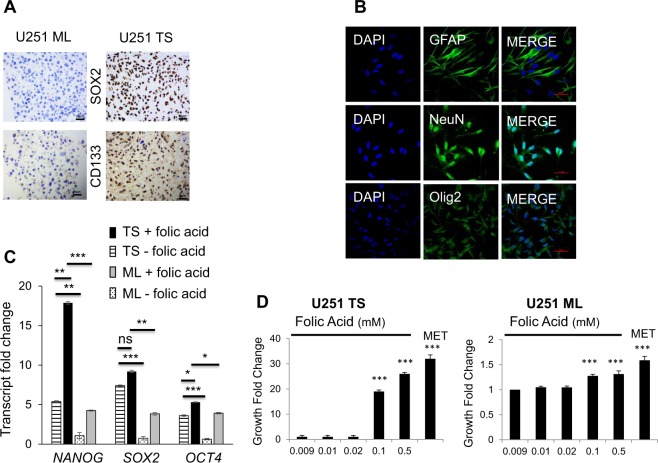
Folic acid promotes U251 tumor spheres stemness. **a** TS overexpress SOX2 and CD133 as shown by immunohistochemistry, *n* = 3. Scale bar = 20 μm. **b** TS differentiate into neurons, oligodendrocytes and astrocytes as indicated by immunofluorescence staining with GFAP, NeuN and OLIG2, respectively, *n* = 3. Scale bar = 10 μm. **c** TS overexpress *NANOG*, *SOX2*, and *OCT*4 transcripts as shown by RT-qPCR. Addition of folic acid (0,5 mM) further increases the expressions of *NANOG, SOX2*, and *OCT4* in TS by comparison with ML; RNA was extracted from 3 independent cell cultures then gene expression was measured by 3 independent RT-qPCR and normalized for the geometric mean of *TBP*, *RPL13a*, and *HMBS*. **d** Folic acid rescues TS formation when added at a concentration ≧ 0.1 mM in methionine-free media. Cells are grown in methionine free DMEM which already contains 0.009 mM folic acid then folic acid is added to reach the indicated final concentrations. The MET sample is used as a reference and represents cells grown in DMEM containing 0.01 mM methionine. Samples are compared to cells grown in 0.009 mM folic acid, *n* = 9; *p < 0.05, **p < 0.01, ***p < 0.001, ns = not significant; ML; monolayer cells, TS: tumor spheres;

Stem-like characteristics of the tumor spheres (TS) derived from glioblastoma (GBM) U251 cells were further verified. Immunocytochemistry, carried out on cross-sectional slices of embedded cell pellets, showed a uniformly stronger staining for both SOX2 and CD133 in TS compared to monolayer (ML) cells (Fig. 4a). SOX2 and CD133 are implicated in the maintenance of self-renewal and tumorigenic potential. When single tumor spheres were dissociated to single cells and subsequently cultured in adherent differentiating condition, these single cells differentiated into NeuN, OLIG2, or GFAP expressing neurons, oligodendrocytes, or astrocytes, respectively (Fig. 4b), thus indicating that U251 TS are capable of multi-lineage differentiation. Furthermore, RT-qPCR (*n* = 3), evidenced higher *SOX2* (7.42 ± 0.11 vs 0.75 ± 0.37, TS vs ML, *p* = 0.001), *NANOG* (5.39 ± 0.18 vs 1.07 ± 0.68, TS vs ML, *p* = 0.02), and *OCT4* (3.61 ± 0.15 vs 0.63 ± 0.15, TS vs ML, *p* = 0.0003) transcription in TS (Fig. 4c). Most importantly, folic acid upregulated the expression of the pluripotent genes in TS and ML (Fig. 4c). In TS, the relative expressions of the pluripotent genes were: *SOX2* 7.42 ± 0.11 (before folic acid addition, TS−) vs 9.19 ± 0.25 (after folic acid addition, TS+) *p* = 0.32; *NANOG* 5.40 ± 0.18 vs 17.86 ± 0.25 (TS− vs TS+; *p* = 0.005), and *OCT4* 3.61 ± 0.16 vs 5.30 ± 0.10 (TS− vs TS+; *p* = 0.04); and in ML, *SOX2* 0.76 ± 0.38 (before folic acid addition, ML−) vs 3.84 ± 0.28 (after folic acid addition, ML+), *p* = 0.007; *NANOG* 1.08 ± 0.68 vs 4.25 ± 0.11, ML− vs ML+, *p* = 0.04, and *OCT4* 0.64 ± 0.16 vs 3.92 ± 0.17, ML− vs ML+, *p* = 0.0003. These results contribute to explain why the addition of folic acid (≥0.1 mM) to a methionine-free medium restored the formation of U251 TS (Fig. 4d) (*n* = 9; *p* = 0.0002) as well as that of the U87 TS, LN299 TS, and T98G TS (Fig. SI 4). Taken together, these data support the finding that U251 TS possess stemness characteristics, and the addition of folic acid promotes the pluripotent gene expression in both TS and ML cells.

### Low folate metabolism is a common feature of glioblastoma tumor spheres

To find out if the downregulated cytosolic folate cycle enzymes disclosed by transcriptome data of U251 cells triggered functional consequences, we used mass spectrometry (LCMSMS) to quantitate folate isoforms 5-methyltetrahydrofolate, 5,10-methenyltetrahydrofolate, and 10-formyltetrahydrofolate, before and after the addition of folic acid (11 microM) and Formate (300 microM) as substrates.

**Fig. 5:**
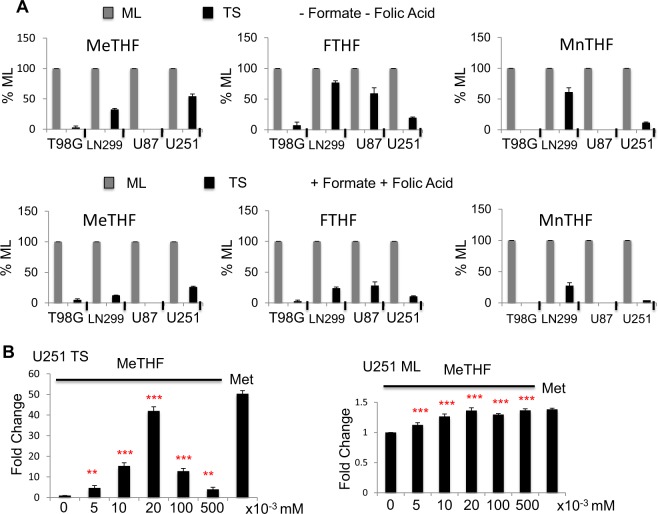
The cytosolic folate cycle is repressed in U251, U87, LN299, and T98G glioblastoma cell lines. **a** Irrespective of folic acid and formate addition, the concentrations of folate isoforms are lower in TS than in ML. In U251 cells, the addition of folate cycle substrates further decreases the concentrations of folate isoforms. This paradoxical response was not detected in all glioblastoma cell lines partly due to low isoform concentrations. Isoform concentrations in TS are expressed as a percentage of the same isoform concentration measured in ML. A value of 100% was arbitrarily attributed to isoform concentration in ML. « + » sign indicates that folic acid and formate were added to the medium and «−» sign that they were not added, *n* = 3. **b** 5-Methyltetrahydrofolate restores U251 TS formation with an optimal concentration at 0.02 mM, *n* = 3. Cells are grown in methionine free media then 5-methyltetrahydrofolate is added to reach the indicated concentrations. The MET sample is used as a reference and represents cells grown in DMEM containing 0.01 mM methionine *p < 0.05, **p < 0.01, ***p < 0.001, MeTHF = 5-methyltetrahydrofolate, FTHF = 10-formyltetrahydrofolate, MnTHF = 5,10-methenyltetrahydrofolate, ML = monolayer cells, TS = tumor spheres

We found that independent of substrates addition, in all 4 GBM cell lines, the levels of all 3 folate isoforms were lower in tumor spheres (TS) than in monolayer cells (ML) (Fig. 5a and Table SI 2). Moreover, the addition of folate substrates led to a tendency of “further” cytosolic folate repression, which reached statistical significance in U251 cells but not in all GBM cells i.e., U251 TS treated with folate substrates had even less folate isoforms than did non-treated TS (Fig. 5a). Precisely, in U251 TS, the levels of folate isoforms varied after the addition of folate substrates as follows: 5-methyltetrahydrofolate, 54.24 ± 3.67% (before) vs 26.14 ± 1.22% (after); 5,10-methenyltetrahydrofolate, 11,64 ± 1.06% (before) vs 4.23 ± 0.30% (after), and 10-formyltetrahydrofolate 19,57 ± 1.26%, (before) vs 10.75 ± 0.87% (after) (for all isoforms, *n* = 4, *p* < 0.001). No such tendency of “further” cytosolic folate repression was observed in ML cells. Taken together, these results indicate that low cytosolic folate cycle activity is a feature of GBM TS.

### 5-Methyltetrahydrofolate restores glioblastoma but not meningioma tumor spheres formation in the absence of methionine

**Fig. 6:**
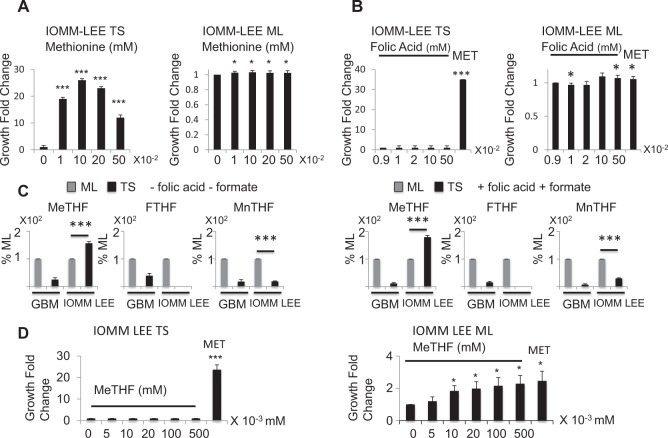
Contrary to the glioblastoma cell lines, folic acid, and 5-methyltetrahydrofolate fail to promote tumor spheres formation in the meningioma cell line IOMM-LEE. **a** IOMM-LEE TS are methionine dependent. Methionine, up to 0.1 mM increases TS growth. Cells are cultured in methionine free media supplemented with the indicated amounts of exogenous methionine. Samples are compared to cells grown with no exogenous methionine addition, *n* = 9. **b** Folic Acid does not rescue IOMM-LEE TS formation in methionine depleted media. Cells are grown in methionine free DMEM which already contains 0.009 mM folic acid, then more folic acid is added to the media to reach the indicated final concentrations. The MET sample is used as a reference and represents cells grown in DMEM containing 0.01 mM methionine. Samples are compared to cells grown in 0.009 mM folic acid, *n* = 9. **c** 5-Methyltetrahydrofolate level is higher in IOMM-LEE TS than ML. 10-Formyltetrahydrofolate and 5,10-methenyltetrahydrofolate exhibit lower concentrations in IOMM-LEE TS compared to IOMM-LEE ML cells, however, 5-methyltetrahydrofolate is more elevated in TS than in ML cells. Addition of folic acid and formate keeps these results unchanged. Folate isoforms are averaged in all 4 glioblastoma cell lines and the mean is represented. « + » sign indicates that folic acid and formate were added to the media and «−» sign that they were not added, *n* = 3. **d** 5-Methyltetrahydrofolate does not restore U251 TS formation, *n* = 3. Cells are grown in methionine free media then 5-methyltetrahydrofolate is added to reach the indicated concentrations. The MET sample is used as a reference and represents cells grown in DMEM containing 0.01 mM methionine. *p < 0.05, **p < 0.01, ***p < 0.001, MeTHF = 5-methyltetrahydrofolate, FTHF = 10-formyltetrahydrofolate, MnTHF = 5,10-methenyl tetrahydrofolate, ML = monolayer cells, TS = tumor spheres

Given that 5-methyltetrahydrofolate is the immediate upstream one-carbon donor for de novo methionine synthesis, we asked whether the decreased 5-methyltetrahydrofolate production in glioblastoma (GBM) tumor spheres (TS) could underlie the reason why exogenous methionine is required for TS formation. Indeed, when 5-methyltetrahydrofolate was added to methionine-free media, it restored TS formation in U251 cells (Fig. 5b) as well as in U87, LN299, and T98G cells (Fig SI 5). Remarkably, the relationship between 5-methyltetrahydrofolate concentration and TS formation is bell-shaped and reminiscent of the relationship between methionine concentration and TS formation. We proceeded to ask if the cytosolic folate cycle repression described for GBM is common to TS derived from other brain cancer cell types and may thus account for their methionine dependency. To do this, we characterized the folate metabolism in IOMM-LEE, a high-grade meningioma cell line. IOMM-LEE TS were found indeed methionine-dependent (Fig. 6a), just like the GBM cell lines, even though the optimal methionine concentration (0.1 mM) was higher than that observed for GBM cell lines. However, unlike in GBM cell lines, Folic Acid did not rescue IOMM-LEE TS formation in methionine-free media (Fig. 6b). Furthermore, we found that the degree of repression of cytosolic folate cycle is different from that of the GBM TS cells. In part similar to GBM, 5,10-methenyltetrahydrofolate and 10-formyltetrahydrofolate were lower in IOMM-LEE TS than in ML however, these lower levels were not further repressed by substrate addition: 5,10-methenyltetrahydrofolate 18.53 ± 1.55% (before substrate addition) vs 30.06 ± 2.27% (after substrate addition), 10-formyltetrahydrofolate was not detectable (either before or after). The most surprising finding in IOMM-LEE cells was that, unlike in GBM cells, 5-MethylTetraHydroFolate level was higher in TS than ML cells (Fig. 6c and Table SI 2): 156.48 ± 6.13% (before substrate addition) vs 179.27 ± 6.71% (after substrate addition), *n* = 3, *p* < 0.001. In concordance with these results, 5-methyltetrahydrofolate did not rescue TS formation in the absence of methionine in IOMM-LEE cells (Fig. 6d). Taken together, these results highlight that, although plastic folate metabolism underlies the methionine dependency of the brain cancer cells, tissue-specific fine-tuning of the folate cycle characterizes the needs of the individual TS to proliferate.

### DNA methylation is only partially responsible for folate cycle enzymes downregulation

To determine if folate cycle enzymes repression is a result of modifications of DNA methylation, we compared genome-wide DNA methylation patterns between U251 TS and ML cells. Our data summarized from 15,037 probes using a FDR (false discovery rate) of <0.01 showed that global methylation profiles remained stable, with only minor changes in both the hypo- and hypermethylated regions (Fig. SI 6). DNA methylation data with significantly altered genes is displayed in Table SI 3. The 2032 hypomethylated and 1880 hypermethylated DMRs were mapped to 3514 unique genes with functions mainly in chromatin remodeling, polycomb silencing, cell cycle, adhesion, cell migration, and neuronal stemness. Many downregulated genes involved in cell cycle have hypermethylated DMRs like *SMAD3* (methylation M = +4%) and *E2F2* (M = +7.9%). Upregulated stemness signature genes with hypomethylated DMRs included *BDNF* (−5.6%), *CLU* (M = −6.3%), *ELF4* (M = −0.44%), *FGF10* (M = −2%) *GFAP* (M = −7%) and *TBX3* (M = −4%). Downregulated stemness genes with hypermethylated DMRs included *FZD7* (M = +2.4%), *ID1* (M = +7.6%), *ID3* (M = +9.3%) and *KLF4* (M = +1.8%).

Among the 139 genes implicated in one-carbon metabolism that were analyzed, only 15 transcripts were significantly negatively correlated. Those which were downregulated and hypermethylated included *ATIC* (M = +0.3%, fold change FC = 0.66), *GGH* (M = +0.8%, FC = 0.33), *GOT1* (M = +0.06%, FC = 0.87), *GOT2* (M = +0.8%, FC = 0.69), *MTHFSD* (M = +0.7%, FC = 0.82), *MTR* (M = +0.2%, FC = 0.74), *MTRR* (M = +0.3%, FC = 0.62), *SHMT1* (M = +0.3%, FC = 0.9), *SMS* (M = +1.2%, FC = 0.55) and *SPR* (M = +0.8%, FC = 0.81). Those which were upregulated and hypomethylated included *AMD1* (M = −0.3%, FC = 1.95), *DHFRL1* (M = −0.3%, FC = 1.3), *GNMT* (M = −3.1%, FC = 1.81), *MTFMT* (M = −0.9%, FC = 1.57) and *MTHFR* (M = −3.7%, FC = 4.00). Taken together, these data incite to investigate other epigenetic modifications than DNA methylation, in order to find out if they are regulators of the folate cycle.

## Discussion

We found that tumor spheres (TS) derived from 4 glioblastoma cell lines (U251, U87, LN299, T98G) and IOMM-LEE meningioma cell line require methionine for growth whereas their corresponding monolayer adherent cells (ML) can grow without methionine. Additionally, the growth of all TS is characterized by a bell-shaped methionine dependency which can possibly be explained by several mechanisms. First, methionine is an allosteric inhibitor of the methionine cycle. An excess methionine decreases de novo methionine synthesis, which in turn reduces total intracellular methionine^17–21^. Based on this, a methionine threshold is expected to exist to stimulate TS growth, and above this threshold level, methionine will inhibit TS growth. Our measurements of SAM and methionine levels after the addition of methionine, 5-MethylTetraHydroFolate, or Folic Acid did not support this hypothesis as the variations of intracellular SAM and methionine were inconsistent with those predicted by allosteric inhibition (Fig. SI 7). Second, methionine in excess can increase the activity of ALDH1L1 and deplete the folate-activated one-carbon pool via catalyzing the conversion of 10-formyltetrahydrofolate to tetrahydrofolate. Increasing ALDH1L1 activity as a result of increasing methionine concentration may thus impairs the TS proliferation via the reduction of available one-carbon units^22^. Moreover, the more SAM produced as a result of increasing methionine, the higher the activity of mTORC1^23^, which in turns block the self-renewal characteristics of the stem cells^24^. This latter effect of SAM highlights the importance of methionine as a signaling metabolite capable of regulation of diverse signaling events.

Our data also revealed that methionine dependency of TS growth differs depending on the tissue origins of the tumor. Here, folic acid at a concentration of ~0.1 mM was shown to promote all GBM TS formation (Fig. 4, Fig. SI 4) but not IOMM-LEE TS formation (Fig. 6b). Similar difference was also observed using 5-methyltetrahydrofolate. This differential responsiveness of the TS growth to either 5-methytetrahydrofolate or folic acid is remarkable in that the TS response to growth actually depends on the relative content of cellular 5-methyltetrahydrofolate—positive response with lower 5-methyltetrahydrofolate in TS vs ML cells (in the case of GBM), and negative response with higher 5-methyltetrahydrofolate in TS vs ML cells (in the case of meningioma). It is worth noting that the threshold concentration of folic acid for GBM TS growth promotion is in the same order of magnitude as that reported in subjects with excessive folic acid intake^25^. Excessive folate intake may thus promote cancer stem cells formation in GBM patients.

Transcriptome-wide sequencing revealed no mutations in genes implicated in either the de novo synthesis or salvage pathway of methionine, thus ruling out an insufficient intracellular methionine as the cause of methionine dependency. This result is consistent with the observation that ML are not sensitive to methionine deprivation, and is compatible also with the hypothesis that methionine dependency can stem from deregulated methionine utilization^26–28^. Furthermore, transcriptome-wide sequencing revealed that the expressions of *DHFR*, *SHMT1*, and *MTHFD1* of the cytosolic folate cycle is downregulated in TS vs ML cells. Methionine dependency of the GBM TS here is thus a consequence of a metabolic reprogramming of the folate cycle and not a consequence of genetic heterogeneity of the parent cells as the same parent cells with non-mutated one-carbon cycle genes were used to produce both the TS and the ML cells. Similar observations have been made in embryonic stem cells, which differentiate if methionine is withdrawn^29^. A role for methionine in the maintenance of pluripotency is supported by the fact that both embryonic and induced pluripotent stem cells express high levels of enzymes participating in methionine metabolism^13^.

Results of the folate isoform measurements in U251 and the 3 other GBM cells corroborated U251 cells transcriptomic data, and supported the idea that a repressed cytosolic folate cycle underlies the methionine dependency of the GBM TS cells. This explains why in the absence of exogenous methionine, no GBM TS formation is possible except with the addition of either 5-methyltetrahydrofolate or folic acid, and why the dependency of GBM TS growth on methionine and on 5-methyltetrahydrofolate bear similar bell-shaped (dose-response) features. Transcriptome data, as well as folate isoform measurements, also suggest that during reprogramming into TS, GBM cells reorient its one-carbon units away from methionine synthesis via folate cycle reprogramming. Altered folate metabolism has indeed been implicated in promoting cancer stem cell formation. For example, low folate was linked to pluripotent gene expression via sonic hedgehog or Akt-mTOR-HIF1-FOXO3a pathways in colorectal^30^ and lung cancer^31^. Similarly, 5-MethylTetraHydroFolate down-regulation was observed during reprogramming of mouse embryonic fibroblasts (MEFs) into mouse induced pluripotent cells (iPSC)^32^. In germline stem cells of the *Caenorhabditis Elegans*, folate related compounds were shown to be necessary for germline stem cell proliferation^33^. Thus, as folates continue to enthuse controversies in (cancer) cell growth, their precise roles may be tissue-dependent and subject likely to the influence of tumor environments.

Our transcriptomic data revealed further that while the transcriptions of the cytosolic folate enzymes are reduced in U251 TS cells, the transcriptions of certain mitochondria folate enzymes are increased. We confirmed these observations with protein expression of SHMT2, and ALDH1L2 using immunocytochemistry (Fig. 3). These results suggest that in GBM TS, mitochondrial folate cycle reorients its one-carbon units away from formate synthesis to the production of NADPH via ALDH1L2. This leads to less (folate-dependent) mitochondria formate production, reduces subsequently the levels of 10-formyltetrahydrofolate, 5,10-methenyltetrahydrofolate and 5-methyltetrahydrofolate in the cytoplasm and brings about the methionine dependency of the GBM TS as a result of insufficient de novo methionine synthesis. On the other hand, NADPH controls the production of reactive oxygen species which are involved in the maintenance of pluripotency^34^. Taken together, these data incite further reconsideration of the therapeutic usefulness of antifolates, based on their specific action on individual folate enzymes, in order to avoid therapy resistance of tumors like GBM: the effect of antifolates on cell growth might differ depending on whether they target mitochondrial or cytosolic folate enzymes^35,36^.

Methylome data indicated that despite an upper limit of 25% of the methylation alterations associated with neural stemness and cell cycle arrest signatures, DNA methylation contributed only minorly to the folate cycle repression. However, this finding cannot rule out epigenetic modifications as regulators of the folate cycle because other mechanisms, like histone modification, RNA methylation,and non-coding RNA, might prove to intervene in controlling folate metabolism.

In conclusion, our data argue for the idea that folate metabolic reprogramming can underlie the transformation of differentiated cancer cells into stem-like cancer cells. The in vitro data shown here suggest that such metabolic alteration may differ depending on the tumor tissue types. GBM and meningioma display a difference in metabolic rewiring which, albeit renders both TS types methionine-dependent, contributes also to their distinct responsiveness to the supplementation of folic acid and 5-methyltetrahydrofoalte for their growth under limited methionine condition. These mechanistic insights, although remaining to be confirmed in vivo, pave the way to such reflections as the risk of excessive folate intakes in the formation of cancer stem-like cells and the pertinence of antifolate drug for the eradication of these cells in patients with glioblastomas and meningiomas.

## Materials and methods

### Cell culture

The U251 and U87 malignant glioblastoma cells used were authenticated by ECACC (European Collection of Authenticated Cell Cultures) (09063001 and 89081402; Sigma Aldrich, Saint Quentin Fallavier, France). The IOMM-LEE cells were a gift from Dr. Gillespie and Dr. Jensen, University of Utah, USA^37^. The Ln229 (CRL-2611), and T98G (CRL-1690) malignant glioblastoma cells were obtained from ATCC. They were routinely screened to make sure they were free from mycoplasma contamination (Venor GEM one step, MB Minerva Biolabs, Berlin, Germany). Monolayer (ML) cells culture was made under adherent culture condition with DMEM-F12 (D6434 Sigma-Aldrich), 10% fetal calf serum (FCS) (Sigma-Aldrich), 1% penicillin–streptomycin (Sigma-Aldrich) at 37 °C, 5% CO_2_. Tumor spheres (TS) were obtained after preparing single cell suspensions and plating cells at a density of 20,000 cells per well in non-adherent 6-well plates coated with 2% 2-hydroxyethylmethacrylate (Sigma-Aldrich). TS medium contained DMEM-F12 (D6434 Sigma-Aldrich) supplemented with 1XB27/0.2 μg/ml EGF/0.2 μg/ml FGF (Fisher Scientific Illkirch Graffenstaden France). Note that the DMEM-F12 used does not contain glutamine but no glutamine was included in neither ML cells nor TS media as no differences in growth were observed with or without glutamine (Fig. SI 8). TS were grown for 7 days in a humidified incubator at 37 °C at an atmospheric pressure with 5% CO_2_. Spheres >50 μm were counted on the 8th day using MoticamX (MoticEurope S.L.U., Barcelona, Spain) and Image J software; these first passage spheres were then dissociated into single-cell suspension for replating again in a new 2-hydroxyethylmethacrylate coated 6-well plate. This process was repeated 2 times and only passage 3 TS were used throughout the study. TS methionine deprivation was tested by the standard limiting dilution assay and in 6-well plates. Both assays yielded similar results. In experiments where methionine concentration needed to be monitored, methionine free DMEM (21013024, Fisher Scientific) was used, supplemented with L-cysteine at 30 mg/L (Sigma-Aldrich), penicillin/streptomycin 1%, and B27/FGF/EGF for TS or 10% FCS for ML (Figs. 1b, 4d, 5b, Fig. SI 5, SI 6). Experiments investigating methionine dependency and the consequence of folic acid and 5-methyltetrahydrofolate addition were performed in triplicate in 3 independent runs.

### Standard limiting dilution assay

An aliquot of U87 cells was diluted in a suitable amount of tumor spheres medium in order to get a concentration of 1 cell per 100 μl of medium. The cell suspension was distributed at 100 μl per well in 96 well-coated plates. 3 entire plates were prepared per condition. Single cell containing wells were checked. Each well containing no cells or 2 or more cells was discarded. The plates were incubated at 37 °C and 5% CO_2_. Every 2–3 days, 50 μl of fresh tumor sphere medium were added. After 10 days, wells were checked for spheres formation. The number of wells containing spheres was divided by the number of total seeded wells and was multiplied by 100.

### RNA extraction and real time PCR

Total RNA was extracted using Trizol^TM^ reagent (Fischer Scientific) following the manufacturer’s recommendation. Reverse transcription was done using the Quantitect Reverse Transcription kit (Qiagen Les Ulis France). Quantitative PCR was performed using SYBR Green I Master Mix buffer (Fischer Scientific) and Quantitect SYBR Green PCR kit (Qiagen). *TBP* (TATA Box Binding Protein), *RPL13a* (Ribosomal Protein) and *HMBS* (Hydroxymethylbilan Synthase) were used as internal standards. Oligonucleotides used are listed in supplemental information. Differences in genes expression were calculated by geometric averaging of multiple internal controls^38^. Relative gene expression was measured in triplicate in 3 independent cell cultures (*n* = 9).

### Immunocytochemistry, western blotting, and fluorescence immunostaining

Immunochemistry was carried out using antibodies against DHFR (1/200, rabbit, ab85056, Abcam Cambridge UK), MTHFD1 (1/1000, rabbit, HPA000704, Sigma-Aldrich), SHMT1 (1/50, rabbit, HPA023314, Sigma-Aldrich), SHMT2 (1/1000, rabbit, GTX125939, Genetex), ALDH1L2 (1/20, rabbit, NPB1–81935, Biotechne), CD133 (1/200, rabbit, ab19898, Abcam) and SOX2 (1/1000, rabbit, 3579s, Ozyme Saint Quentin en Yvelines France). For western blotting, the antibodies used against DHFR (1/700), SHMT1 (1/500), MTHFD1 (1/500) were the same as those used in immunocytochemistry. Beta-actin and alpha-tubulin served as a protein loading control (1/7000, ab197277, Abcam and 1/10000, ab40742, Abcam). For more details, please refer to the sections in supplemental information.

To induce differentiation, single tumor spheres were pelleted, seeded under adherent conditions and maintained in DMEM-F12 supplemented with 1% FCS for 10 days, probed with GFAP (1/200, mouse, MAB360 Sigma-Aldrich), NeuN (1/200, mouse, MAB 377, Sigma-Aldrich), and Olig2 (1/200, mouse, MAB N50, Sigma-Aldrich), washed and incubated with secondary antibody then fluorescence immunostaining was examined under a Nikon C2 microscope (Nikon Amsterdam, Netherlands) to assess cell lineages. All experiments were performed on 3 independent cell cultures.

### LCMSMS determination of folate isoforms

Folate isoforms including 5-methyltetrahydrofolate, 10-formylTHF and methenylTHF, were measured using LCMSMS (Acquity UPLC, Waters Saint Quentin en Yvelines France) equipped with a triple quadripole mass spectrometer (4000 QTRAP, AB Sciex Villebon sur Yvette France). These measurements were performed in ML cells and TS supplemented with the addition of folate substrates, including folic acid (11 μM) and Formate (300 μM). Folates concentrations (nmol/L) were normalized to total proteins concentrations (g/L) and results were expressed in nmol/g protein. Measurements were performed in 3 independent cell cultures. Folate isoform measurements were normalized relative to those of the ML cells taken as 100%, i.e., folate isoform concentrations in TS were expressed as a percentage of folate isoforms concentrations in ML cells.

### RNA sequencing

The data pertaining to RNA sequencing and methylome have been deposited in NCBI’s Gene Expression Omnibus^39^ and are accessible through GEO Series accession number GSE117544. In addition, a secure token has been created to allow the review of the record while it remains in private status and can be requested from the authors.

RNA was extracted from TS and ML cells using Trizol (Fischer Scientific). RNA concentration was measured on Nanodrop 2000 and RNA quality was checked by capillary electrophoresis with PicoRNA chip on Bioanalyzer 2100 (Agilent Les Ulis France). RNA was fragmented and converted to library using NEBNext® Small RNA Library kit (NEB Evry France). DNA library quality was assessed with a High Sensitivity DNA chip on a Bioanalyzer 2100. Library quantification was done using a fluorometer (Fisher Scientific). Libraries were multiplexed and subjected to high-throughput sequencing by using Illumina MiSeq for paired-end read. Data were generated from 3 independent TS cultures and 3 ML cell cultures and analyzed according to the tools detailed in supplemental information.

### Transcriptome analyses

Prior to the transcriptomic study, as the composition of the culture media for TS and ML cell growth were different, we evaluated the content of the amino acids presented in these media using chromatography method to make sure that any difference observed at the level of transcriptome was not due to differences in amino acid contents of the media. We noted no significant differences (data not shown), and proceeded to analyze the transcriptome SEQ in U251 TS vs ML cells (*n* = 3). As criteria, for all transcripts, a false discovery rate (FDR) at a *p*-value of ≤ 0.01 and a change in expression of ≥ 1.5-fold were adapted. For further detailed descriptions and analyses, please refer to Bioinformatics in supplementary information.

### Methylome

Based on the manufacturer’s recommendations, DNA was extracted from tumor spheres and adherent cells using Machery Nagel kit. Then, 1 μg DNA was treated with Zymo kit for bisulfite conversion, and 600 ng of treated DNA were processed following the 450k Human methylation Illumina assay protocol. Data were analyzed according to the tools detailed in supplemental information.

### Statistical analysis

Data generated by cell experiments were analyzed with ANOVA and Student’s *t*-test and were presented as mean ± standard error of the mean (s.e.m). All the reported *p*-values are two-sided, and *p*-values < 0.05 are considered statistically significant. Statistical analyses were performed with Statview 4.0 software (SAS Institute Inc., SAS Campus Drive, Cary NC 27513, USA). For transcriptome and methylome data, differential expression *p*-values were obtained using a two way moderated *t*-test and adjusted for false discovery rate (FDR) following the Benjamini–Hochberg procedure.

More details regarding the Materials and methods can be found in supplementary information.

## Supplementary information

Manuscript Supplemental Information

Supplemental Figure SI1

Supplemental Figure SI2

Supplemental Figure SI3

Supplemental Figure SI4

Supplemental Figure SI5

Supplemental Figure SI6

Supplemental Figure SI7

Supplemental Figure SI8

Supplemental Table SI1

Supplemental Table SI2

Supplemental Table SI3
