# Supplementary material for: Folate can promote the methionine-dependent reprogramming of glioblastoma cells towards pluripotency
Source: Cell Death Dis. 2019 Aug 8;10(8):596. doi: 10.1038/s41419-019-1836-2 (PMC6687714; doi:10.1038/s41419-019-1836-2)
Supplement: Supplementary file 1 — Manuscript Supplemental Information [file 41419_2019_1836_MOESM1_ESM.docx]

**Supplemental information**

**RNA extraction and Real time PCR.**

Amplification reactions for qPCR were run on iCycler (Bio-Rad Marnes-la-Coquette France): Taq DNA polymerase was activated at 95°C/15 min; amplification was performed in 40 cycles (denaturation at 94°C/10s, annealing 57°C or 60°C or 62°C /15s, extension at 72°C/15s). The oligonucleotides (Eurogentec Seraing Belgium) used were as follows:

NANOG S : 5’-ATGCCTCACACGGAGACTGT-3’AS : 5’-AGGGCTGTCCTGAATAAGCA-3’

SOX2 S : 5’-GAAAAACGAGGGAAATGGG-3’AS : 5’GCTGTCATTTGCTGTGGGT-3’

OCT4 S : 5’-AACGACCATCTGCCGCT-3’AS : 5’CGATACTGGTTCGCTTTCTCT-3’

TBP S : 5’-TTGGGTTTTCCAGCTAAGTTCT-3’AS : 5’CCAGGAAATAACTCTGGCTCA-3’

RPL13a S : 5’-CAAGCGGATGAACACCAAC-3’AS : 5’-TGTGGGGCAGCATACCTC-3’

HMBS S : 5’-TGTGGTGGGAACCAGCTC-3’AS : 5’-TGTTGAGGTTTCCCCGAAT-3’

**Immunocytochemistry**

Paraffin sections (5μm) containing PAF fixed U251 adherent or tumor spheres were immersed in a 10mM sodium citrate buffer (pH 6) for 20 min at 97 °C for deparaffinization and antigen retrieval. Immunochemical staining was performed following the manufacturer’s recommendations on a Dako Autostainer Plus (DakoCytomation Glostrup Denmark) with a Flex+ Envision revelation system (Dako Cytomation) and either a rabbit or mouse monoclonal antibody (DHFR, MTHFD1, SHMT1, CD133, SOX2.) using biotin-streptavidin amplification and diaminobenzidine as a chromogen.

**Total protein extraction and Western blot analysis:**

Total proteins were extracted with a solution containing sodium phosphate anhydrous dibasic, potassium dihydrogen phosphate, 150mM NaCl, 1% Nonidet P40, 0.5% sodium deoxycholate, sodium dodecyl sulfate, and Complete Protease Inhibitors (Sigma-Aldrich) after washing twice with ice-cold 1X PBS. Lysates were then subjected to a thermal shock followed by a centrifugation at 12,000 rpm for 30 min. The protein concentration of the supernatant was determined using BCA Protein Assay kit (Fisher Scientific) and BSA as standard protein. In general, 20 ug of the total proteins were loaded per lane for SDS-PAGE. The stacking and the separating gel contained 10% of acrylamide. Proteins were electrotransferred onto PVDF or Nitrocellulose membranes (Sigma-Aldrich) in 25mM Tris buffer containing 192mM glycine and 20% (v/v) methanol. The membranes were then blocked either with 5% non-fat milk or 5% BSA for 1 h at room temperature. The membrane was then incubated overnight with various primary antibodies: anti-DHFR, anti-SHMT1, and anti-MTHFD1. Equal protein loading was confirmed using anti-beta-actin. Appropriate secondary antibodies conjugated to HRP were used for detection with ECL or ECL PLUS reagent (Sigma-Aldrich). Density plots were constructed after normalization with the loading control actin. Images were analyzed with the Image J software.

**Immunofluorescence**

Suspension of single tumor sphere were seeded on coverslips deposited on the bottom of wells in a 24-well plate. Differentiation was induced by maintaining the trypsinized TS cells in DMEM-F12 medium supplemented with 1% FCS for 10 days. Cells were then fixed with paraformaldehyde for 10 min at 4°C, permeabalized with 0.3% tritons, and then blocked with BSA for 1 h and incubated overnight with the primary antibodies: GFAP, NeuN, and OLIG2 in a humid chamber at 4°C under gentle agitation. Coverslips were washed four times in BSA/PBST for 5 min then incubated with the fluochrome-labeled secondary antibody for 1 h at room temperature under gentle agitation. Nucleus were counertstained with 4, 6-diamidino-2-phenylindole (DAPI) (0.43 mg/ml in PBS; Sigma Aldrich, Saint Quentin Fallavier, France). Coverslips were washed four times in PBS and mounted using a minimal volume of fluoromount medium (DAKO, Carpinteria, CA, USA). The immunostained cells were imaged with 60X oil immersion lens, using Nikon C2 equipped with three laser lines (405, 488 and 543 nm).

**LCMSMS determination of folate isoforms**

For LCMSMS measurements, both adherent and tumor sphere cells were cultured in the presence of methionine-containing media. However, to minimize possible transcriptional, translational, as well as allosteric effects of methionine on folate enzyme activity, culture media for both cells types were switched during a period of 24h to methionine-free media (containing otherwise normal amounts of other ingredients), then folic acid and formate were added for 24h. Adherent or tumor spheres were collected after washing with 1X PBS. The cell pellets were simultaneously suspended and lysed through 26g needles in a 500uL aliquot of 0.1X PBS containing 100 mM DL-Dithiothreitol (DTT) and 25 mM ascorbic acid. The cell lysates were then centrifuged for 30 min at 20,000 g and folates isoforms were measured in the supernatant.

**Bioinformatics**

**RNA-Seq pipeline and tools**

Raw paired-end sequencing data corresponding to 100-nucleotide-long reads was compiled in FASTQ files complying the format standard.

Quality control of sequence reads was done using FastQC v0.11.5 (http://www.bioinformatics.babraham.ac.uk/projects/fastqc/). Generic Illumina Truseq adapters were removed with cutadapt v1.11 ^1^, discarding trimmed reads shorter than 30 nucleotides, with parameters « -a AGATCGGAAGAG -A AGATCGGAAGAG -m 30 --no-indels -O 5 ».

Read mapping and main filtering were performed using HISAT2 v2.0.4 ^2^ -- a tool which takes into account and allows for splicing site discovery on the fly -- against reference genome hg38 as described below, with a reference index built to account for population SNPs as well as known transcripts. Such an index can be found here: ftp://ftp.ccb.jhu.edu/pub/infphilo/hisat2/data/grch38_snp_tran.tar.gz. Only properly paired and uniquely mapped reads were retained, with additional scoring constraints applied: « --score-min L,0,-0.2 --sp 10,3 --no-unal --dta --no-mixed ». Samtools v1.3.1 ^3^ were used for manipulating the alignment files throughout the downstream analysis. PCR duplicates were flagged with Picard v1.13 MarkDuplicates (https://broadinstitute.github.io/picard/).

Differentially spliced transcripts were assembled from the obtained alignments with stringtie v1.3.3b ^4^. Our method took advantage of the proposed workflow for identifying known as well as novel isoforms (https://ccb.jhu.edu/software/stringtie/index.shtml?t=manual), using an annotation file for hg38 in gtf format as a guide (see details below). The following parameters were used: first step is applied for each sample « -f 0.2 -j 3 -c 10 -M 0.5 » ; second step merges all transcripts of all samples « --merge -m 200 » ; and the last step estimates abundances and read coverage for all the merged transcripts, for each sample « -A -C -f 0.2 -j 3 -c 10 -M 0.5 ». Two tables were generated from these results, one compiling raw read counts at gene level, and another one at transcript level. Both files were fully annotated with known symbols corresponding to gene and transcript genomic locations, whenever possible.

Raw count tables were filtered by applying a minimum expression threshold for a gene or transcript. Those should be expressed (non-zero value) in at least two samples, and present an average expression value across all samples higher than 1/10.000.000 of the average library size. Data was further adjusted with the TMM normalization method ^5^, and finally was log2- and cpm- (count per million) transformed -- following good-practice measures for assessing differential expression -- as described before ^6^.

**Downstream transcriptomics**

Differential expression p-values were obtained using a two way moderated t-test and adjusted for false discovery rate (FDR) following the Benjamini–Hochberg procedure.

All hierarchical clusterings were carried out with Cluster 3.0 ^7^ with gene-median-centered values, using uncentered Pearson’s correlation as similarity metric. Corresponding heatmaps were obtained with a customized version Slcview derived from latest version 1.1.2 (http://slcview.sourceforge.net/). For targeted clusterings, gene sets were downloaded from MSigDB (http://software.broadinstitute.org/gsea/msigdb), section C2, and compiled from either KEGG, BioCarta, or REACTOME curated gene sets.

Functional annotations were performed using Gene Ontology (GO) ^8^ and GoMiner ^9^ on selected gene sets. Enrichment of GO terms was determined as follows: Enrichment = frequency of the GO term in the gene selection/frequency of the GO term in the complete set of expressed genes. Resulting FDRs were computed by bootstrapping the gene sets 50 times, and was used to define a significance threshold (FDR < 0.01)

KEGG pathway analyses (www.genome.jp/kegg/pathway.html) were carried on with gage ^10^. When used, rendering of the most significant/disturbed pathways was achieved with the R Pathview package ^11^ with a set selective set of significant genes (p-value < 0.01) and their respective plain fold changes (FC > 1: red, overexpressed ; FC<1: green, underexpressed).

**DNA methylation pipeline and tools**

Raw data corresponding to Methylated/Unmethylated channels was extracted from Illumina idat files, normalized according to the FuncNorm procedure ^12^, and transformed into beta-values with the R minfi package ^13^. Beta-values were used for direct interpretation and graphical representation, while M-values were favored for downstream statistics and computations. Probes were further filtered according to their relative positions from known SNPs. To achieve this, the rmSNPandCH function from the R package DMRcate ^14^ was used with dist=3, mafcut=0.05, rmcrosshyb=TRUE as main settings. On the 485.512 unfiltered probes, 435.786 remained.

Empirical Bayesian methods applied on linear models were used to assess for statistical significance of probe differential methylation. Those methods were provided by the R package limma ^15^. P-values were adjusted for FDR following the Benjamini–Hochberg procedure. Differentially methylated region (DMR) computation was accomplished with the dmrcate function, with the parameters: lambda=1000 and C=2. FDR cutoff for first allowing a probe to define a DMR was set to 0.05.

**Annotations**

All transcriptomic analyses were performed using the lastest assembly of the human genome (hg38) as a reference. All gene and transcript annotations were therefore based on the Ensembl ^16^ GRCh38 databases, version 88. Those were mainly taken from ftp://ftp.ensembl.org/pub/release-88/mysql/homo_sapiens_core_88_38/ and ftp://ftp.ensembl.org/pub/release-88/mysql/homo_sapiens_funcgen_88_38/ for local installation and access. The GTF file used during the isoform reconstruction process can be found here: ftp://ftp.ensembl.org/pub/release-88/gtf/homo_sapiens/Homo_sapiens.GRCh38.88.gtf.gz. Once Stringtie had finished the transcript assembly, gene symbols were assigned to returned Ensembl or Stringtie generated IDs based on overlapping positions with Ensembl known transcripts.

Methylome data were analysed using the available Illumina 450k platform annotations, which are strongly relying upon the hg19 assembly. As several tools like minfi and DMRcate still use those platform manifests by default, resulting annotations were lifted to hg38 coordinates as an after-process.

The R package biomaRt ^17^ was useful wherever annotation symbols or genomic locations needed to be converted into appropriate features for program-specific usage or to achieve data integration.

**Figure Legends**

**Figure SI1. Whole transcriptome analysis between MonoLayer (ML) and Tumor Spheres (TS) U251 cells reveals a two-fifth drastic change in their expression profile**. (a) **Left**, hierarchical clustering of the 23048 expressed genes separates two significant clusters of co-expressed genes. Resulting heatmap is represented with downregulated, upregulated and median genes in blue, yellow and black, respectively. Associated fold changes are plotted in blue dots and modeled with a red smoothing spline running against the transcriptome**. Middle**, summary of the most relevant biological, cellular and molecular functions for the two significant under- (4515 genes implicated in cell cycle and mitosis processes) and over-expressed (4756 genes mostly implicated in neuronal processes) signatures. Full functional annotations are available in supplementary tables X1 and X2. **Right**, detailed numbers of up and down-regulated genes in TS and their repartition by fold-change classes, 84 and 21 genes show a fold change >= 50 in TS and ML cells, respectively.

**Figure SI2. U251 tumor spheres exhibit cell cycle signatures.** Major downregulation of thousand of genes impacts all aspects of cell growth and mitosis in TS. Circle diagram of the mitosis cell cycle with selected functional annotations including Gene Ontology (GO) families and associated gene enrichments. In bold between brackets: number of downregulated genes / number of genes in the corresponding GO category. Checkpoints are indicated by blue ovals and genes regulating checkpoints in blue text. Genes are represented in capital letters along with positive (arrow up) or negative (arrow down) fold change values. Cyclins, cyclin kinases and cyclin activated stages are shown in red. Histone related functional annotations are showed in orange. All FDR (False Discovery Rate) statistics for functional annotations and gene expressions are significant (<0.01). * Functional annotations are showed and valid for both G1 and G2 phases. ** CDK1 is active in the late stage of phase S and inactive during mitosis. *** CDK2 is mostly active during post-checkpoint G1 and early S phases ;

**Figure SI3. Histone genes are repressed in U251 “tumor spheres”**. **(A)** hierarchical clustering reveals more than 50 histone genes are down-regulated in « tumor spheres ». Resulting heatmap is represented with downregulated, upregulated and median genes in blue, yellow and black, respectively. **(B)** Two large regions encompassing cluster HIST1 (6p21-6p22, over 600 kb) encoding histones H1, H2A, H2B, H3 and H4 and cluster HIST2 (1q21, over 150 kb) encoding histones H2A, H2B and H3 are nearly entirely silenced. Interestingly, each cluster harbors an upregulated «long intergenic non coding RNA » (lincRNA): linc00240 in cluster HIST1 chromosome 6, fold change= 2.31 and linc00869 in cluster HIST2 chromosome 1, fold change= 2.29. Green dots represent downregulated genes, red dots uregulated genes and black dots median genes.

**Figure SI4. Folic Acid promotes tumor spheres growth in LN299, U87 and T98G glioblastoma cell lines when added at a concentration ≧ 0.1 mM in a methionine-free media**. Cells are grown in methionine free DMEM which already contains 0.009 mM folic acid then folic acid is added to reach the indicated final concentrations. The MET sample is used as a reference and represents cells grown in DMEM containing 0.01 mM methionine. Samples are compared to cells grown in 0.009 mM folic acid, n=9. *p<0.05, **p <0.01, ***p<0.001, ML: Monolayer Cells, TS: Tumor Spheres;

**Figure SI5**. **Incremental amounts of 5-MethylTetraHydroFolate exogenously added to the culture medium results in enhanced TS formation in LN299, U87 and T98G glioblastoma cell lines**. Cells were grown in methionine free media then 5-methylTetraHydroFolate was added to reach the indicated concentrations. The MET sample is used as a reference and represents cells grown in DMEM containing 0.01 mM methionine *p<0.05, **p<0.01 ***p<0.001, MeTHF= 5-MethylTetraHydroFolate, FTHF= 10-FormylTetraHydroFolate, 5,10-MnTHF= MethenylTetraHydroFolate, ML= MonoLayer Cells, TS= Tumor Spheres

**Figure SI6. Circos plot of genome-wide DNA methylation changes between adherent cells and tumor spheres**. Outside to inside: A) Chromosome organization with ideograms. The centromeres are in red. B) « Rainfall" plot showing DMRs methylation status. Globally hypermethylated (1880 regions) and hypomethylated (2032 regions) regions are shown in red and green, respectively. Distance between 2 successive DMRs is plotted in a logarithmic scale. C) Genomic densities of hypomethylated (green track) and hypermethylated (red track) DMRs. D) « Rainfall" plot showing DMRs linked to the promoter regions controlling key genes of the cell cycle (black asterisks), U251 tumor spheres stemness signature (blue crossed squares) and one-carbon metabolism (purple diamonds). E) Genomic density (in yellow) of 4582 promoter regions showing correlations between methylome and transcriptome changes. At least 3 probes must correlate negatively (with r <= -1/3) between their methylation and the expression level for a given transcript (Murphy et al., 2013). F) Genomic density of all known and annotated transcripts (grey and innermost track), for comparison with correlation densities in E.

**Figure SI7.** Intracellular concentrations of methionine (MET) and S-AdenosylMethionine (SAM) in U251 cells supplemented with exogenous MET (A), Folic Acid (B) or 5-MethylTetraHydroFolate (MeTHF) (C) exhibited non significant variations preventing from drawing conclusions regarding the effect of allosteric regulators on the methionine cycle.

**Figure SI8. Glutamine does not affect U251 cell growth. (A)** addition of 2 mM glutamine does not change growth rate either for ML cells or TS. ML cells were cultured with DMEM-F12 (D6434 Sigma-Aldrich), 10% Fetal Calf Serum, 1% penicillin-streptomycin. TS were cultured on 2-hydroxyethylmethacrylate coated plates in DMEM-F12 (D6434 Sigma-Aldrich) supplemented with 1XB27/ 0.2 μg/ml EGF/ 0.2 μg/ml FGF. Note that the DMEM-F12 used does not contain glutamine. Growth was compared between cells maintained in DMEM-F12 with no glutamine and DMEM-F12 to which 2 mM glutamine was added exogenously, n=3 **(B)** Glutamine has no impact on *NANOG, SOX2* and *OCT4*, either in ML cells or TS. RNA was extracted from 3 independent cell cultures then gene expression was measured by 3 independent RT-qPCR and normalized for the geometric mean of TBP, RPL13a and HMBS. ML=MonoLayer Cells, TS= Tumor Spheres

**Table SI1** **RNA sequencing data with1.5 fold altered genes**

**Table SI2 Measurements of folate isoforms by mass spectrometry**. Isoform concentrations in TS are expressed as a percentage of isoform concentration in ML. A value of 100% was arbitrarily attributed to isoform concentration in ML. The sign + in table A denotes addition of folic acid and formate to the culture medium and the sign – in table B no addition of folate substrates. GBM cell lines averaged represent the mean of isofom concentrations in all 4 GBM cell lines. ML= Monolayer cells, TS= Tumor Spheres, GBM= Glioblastoma

**Table SI3** **DNA methylation data with the significantly altered genes**,

**Supplementary references**

1 Didion, J. P., Martin, M. & Collins, F. S. Atropos: specific, sensitive, and speedy trimming of sequencing reads. *PeerJ* **5**, e3720, doi:10.7717/peerj.3720 (2017).

2 Kim, D., Langmead, B. & Salzberg, S. L. HISAT: a fast spliced aligner with low memory requirements. *Nat Methods* **12**, 357-360, doi:10.1038/nmeth.3317 (2015).

3 Li, H. *et al.* The Sequence Alignment/Map format and SAMtools. *Bioinformatics* **25**, 2078-2079, doi:10.1093/bioinformatics/btp352 (2009).

4 Pertea, M. *et al.* StringTie enables improved reconstruction of a transcriptome from RNA-seq reads. *Nat Biotechnol* **33**, 290-295, doi:10.1038/nbt.3122 (2015).

5 Robinson, M. D. & Oshlack, A. A scaling normalization method for differential expression analysis of RNA-seq data. *Genome Biol* **11**, R25, doi:10.1186/gb-2010-11-3-r25 (2010).

6 Law, C. W., Chen, Y., Shi, W. & Smyth, G. K. voom: Precision weights unlock linear model analysis tools for RNA-seq read counts. *Genome Biol* **15**, R29, doi:10.1186/gb-2014-15-2-r29 (2014).

7 de Hoon, M. J., Imoto, S., Nolan, J. & Miyano, S. Open source clustering software. *Bioinformatics* **20**, 1453-1454, doi:10.1093/bioinformatics/bth078 (2004).

8 Ashburner, M. *et al.* Gene ontology: tool for the unification of biology. The Gene Ontology Consortium. *Nat Genet* **25**, 25-29, doi:10.1038/75556 (2000).

9 Zeeberg, B. R. *et al.* GoMiner: a resource for biological interpretation of genomic and proteomic data. *Genome Biol* **4**, R28 (2003).

10 Luo, W., Friedman, M. S., Shedden, K., Hankenson, K. D. & Woolf, P. J. GAGE: generally applicable gene set enrichment for pathway analysis. *BMC Bioinformatics* **10**, 161, doi:10.1186/1471-2105-10-161 (2009).

11 Luo, W. & Brouwer, C. Pathview: an R/Bioconductor package for pathway-based data integration and visualization. *Bioinformatics* **29**, 1830-1831, doi:10.1093/bioinformatics/btt285 (2013).

12 Fortin, J. P. *et al.* Functional normalization of 450k methylation array data improves replication in large cancer studies. *Genome Biol* **15**, 503, doi:10.1186/s13059-014-0503-2 (2014).

13 Aryee, M. J. *et al.* Minfi: a flexible and comprehensive Bioconductor package for the analysis of Infinium DNA methylation microarrays. *Bioinformatics* **30**, 1363-1369, doi:10.1093/bioinformatics/btu049 (2014).

14 Peters, T. J. *et al.* De novo identification of differentially methylated regions in the human genome. *Epigenetics Chromatin* **8**, 6, doi:10.1186/1756-8935-8-6 (2015).

15 Ritchie, M. E. *et al.* limma powers differential expression analyses for RNA-sequencing and microarray studies. *Nucleic Acids Res* **43**, e47, doi:10.1093/nar/gkv007 (2015).

16 Aken, B. L. *et al.* The Ensembl gene annotation system. *Database (Oxford)* **2016**, doi:10.1093/database/baw093 (2016).

17 Drost, H. G. & Paszkowski, J. Biomartr: genomic data retrieval with R. *Bioinformatics* **33**, 1216-1217, doi:10.1093/bioinformatics/btw821 (2017).
