# Supplementary figures and images for: Folate can promote the methionine-dependent reprogramming of glioblastoma cells towards pluripotency
Source: Cell Death Dis. 2019 Aug 8;10(8):596. doi: 10.1038/s41419-019-1836-2 (PMC6687714; doi:10.1038/s41419-019-1836-2)

## Slide 1
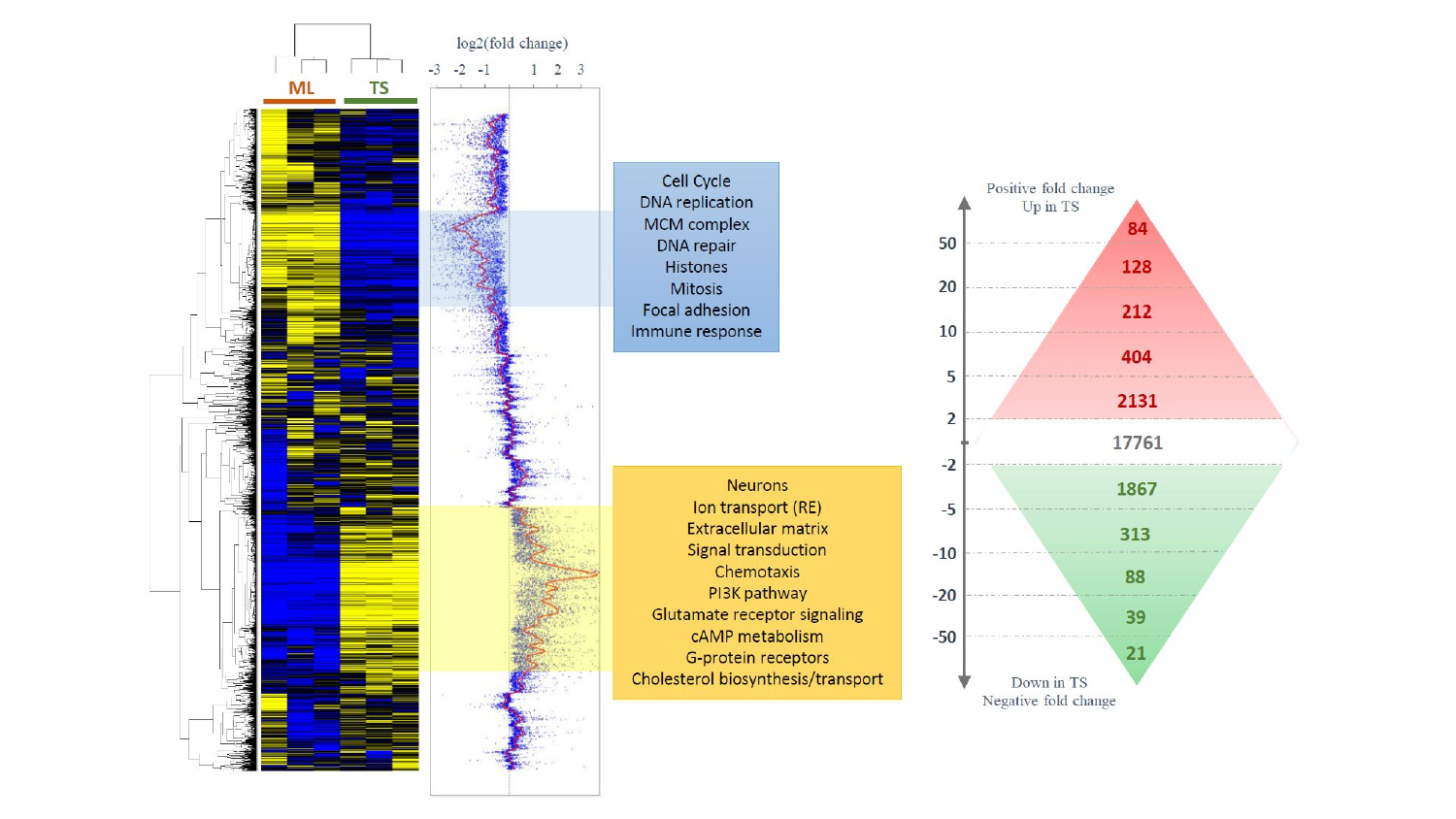

Supplement: Supplementary file 2 — Supplemental Figure SI1 [file 41419_2019_1836_MOESM2_ESM.pptx]

## Slide 1
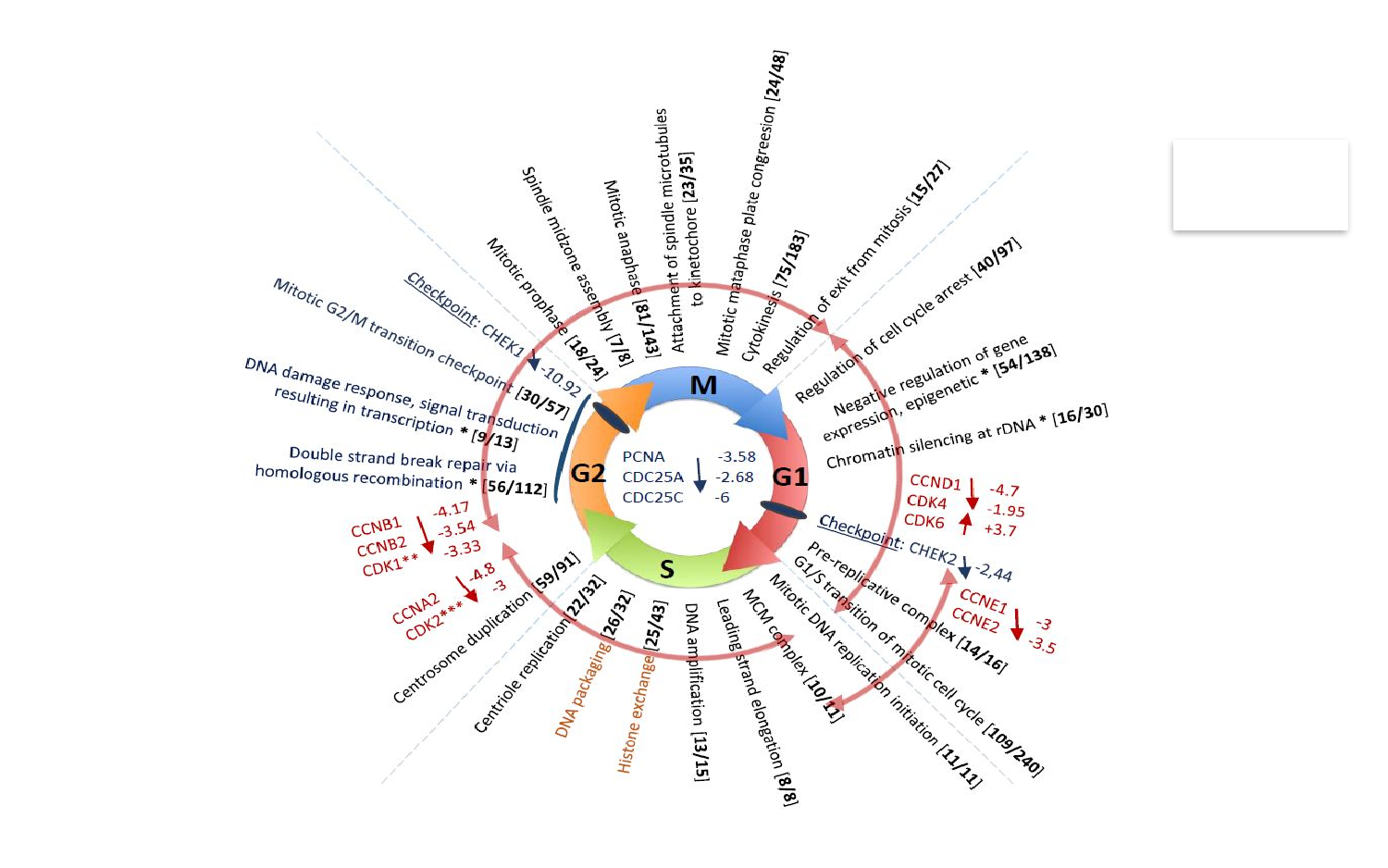

Supplement: Supplementary file 3 — Supplemental Figure SI2 [file 41419_2019_1836_MOESM3_ESM.pptx]

## Slide 1
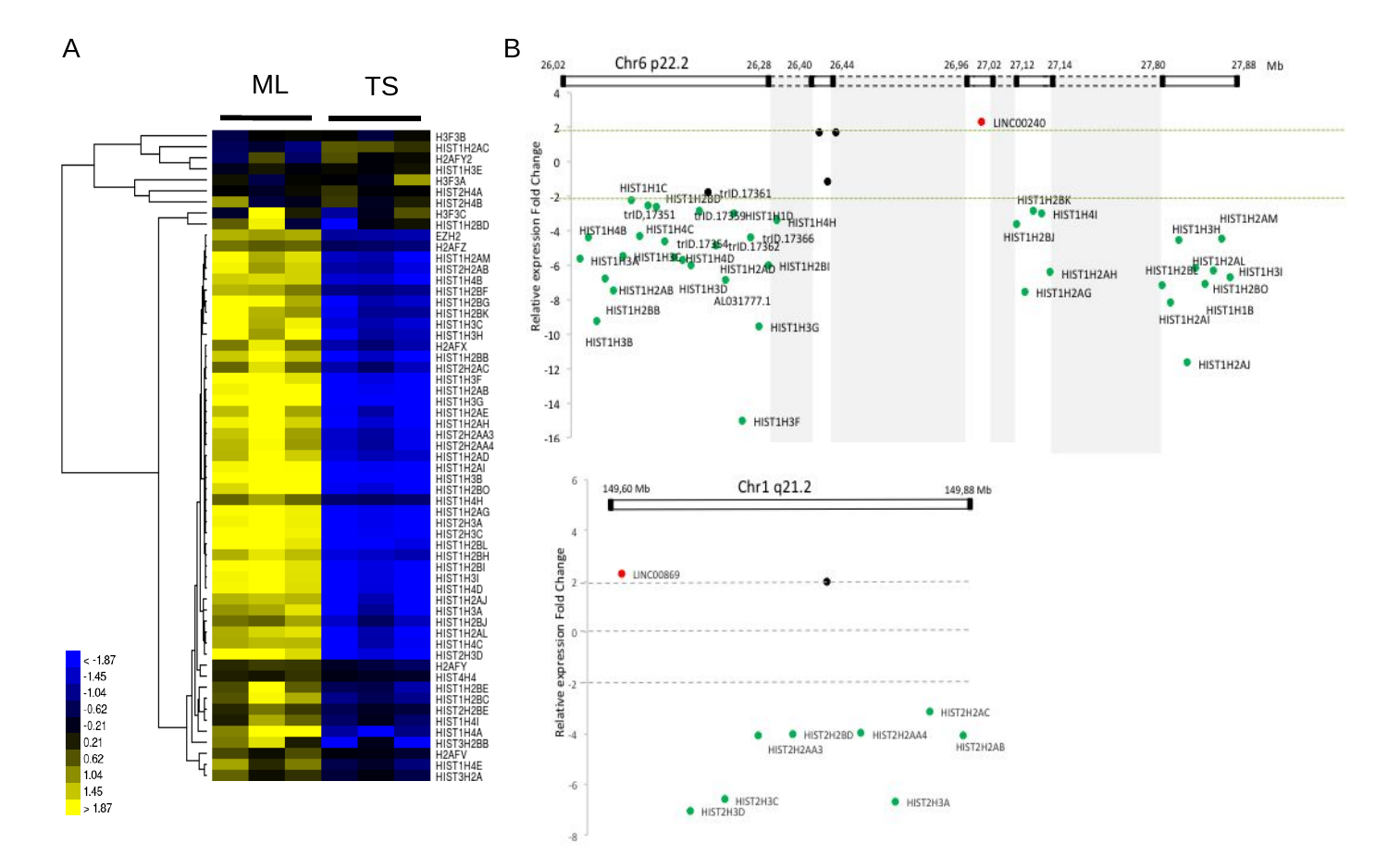

A
B
ML
TS

Supplement: Supplementary file 4 — Supplemental Figure SI3 [file 41419_2019_1836_MOESM4_ESM.pptx]
