# Supplementary material for: Folate can promote the methionine-dependent reprogramming of glioblastoma cells towards pluripotency
Source: Cell Death Dis. 2019 Aug 8;10(8):596. doi: 10.1038/s41419-019-1836-2 (PMC6687714; doi:10.1038/s41419-019-1836-2)
Supplement: Supplementary file 5 — Supplemental Figure SI4 [file 41419_2019_1836_MOESM5_ESM.pptx]

## Slide 1
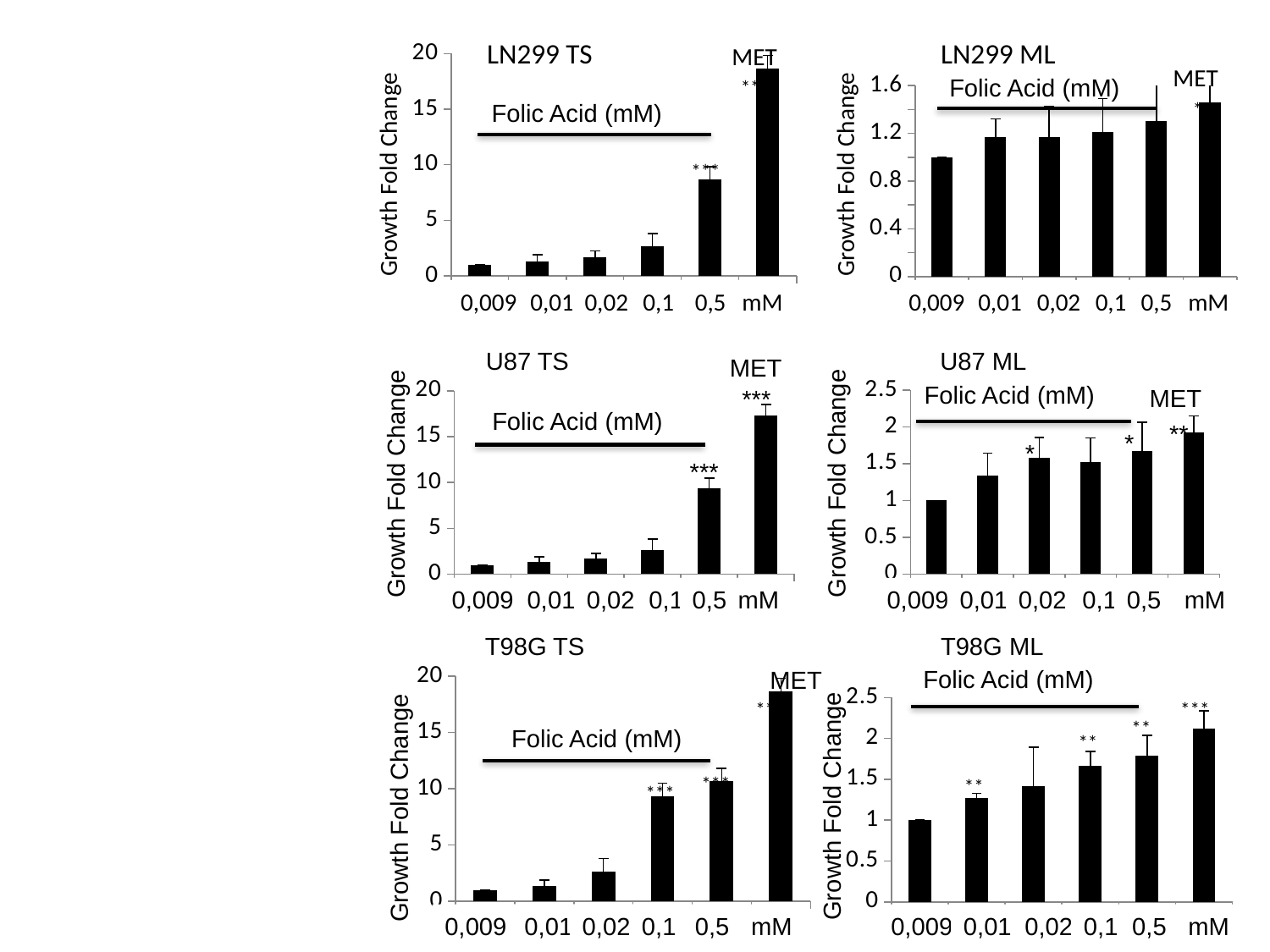

LN299 TS
LN299 ML
MET
### Chart
| Category | |
|---|---|
| 0,009 | 1.0 |
| 0,01 | 1.333333333333333 |
| 0,02 | 1.666666666666667 |
| 0,1 | 2.666666666666666 |
| 0,5 | 8.66666666666667 |
| met | 18.66666666666667 |MET
Folic Acid (mM)
### Chart
| Category | |
|---|---|
| 0,009 | 1.0 |
| 0,01 | 1.172222222222222 |
| 0,02 | 1.172222222222222 |
| 0,1 | 1.211988304093567 |
| 0,5 | 1.306432748538012 |
| met | 1.462573099415205 |***
*
Folic Acid (mM)
***
Growth Fold Change
Growth Fold Change
0,009
0,01
0,02
0,1
0,5
mM
0,009
0,01
0,02
0,1
0,5
mM
U87 TS
U87 ML
MET
### Chart
| Category | |
|---|---|
| 0,009 | 1.0 |
| 0,01 | 1.33888888888889 |
| 0,02 | 1.577777777777778 |
| 0,1 | 1.522222222222222 |
| 0,5 | 1.672222222222222 |
| met | 1.922222222222222 |
### Chart
| Category | |
|---|---|
| 0,009 | 1.0 |
| 0,01 | 1.333333333333333 |
| 0,02 | 1.666666666666667 |
| 0,1 | 2.666666666666666 |
| 0,5 | 9.333333333333332 |
| met | 17.33333333333328 |Folic Acid (mM)
MET
***
Folic Acid (mM)
**
*
*
***
Growth Fold Change
Growth Fold Change
U87 ML
0,009
0,01
0,02
0,1
0,5
mM
0,009
0,01
0,02
0,1
0,5
mM
T98G TS
T98G ML
Folic Acid (mM)
### Chart
| Category | |
|---|---|
| 0,009 | 1.0 |
| 0,01 | 1.333333333333333 |
| 0,02 | 2.666666666666666 |
| 0,1 | 9.333333333333332 |
| 0,5 | 10.66666666666667 |
| met | 18.66666666666667 |MET
### Chart
| Category | |
|---|---|
| 0,009 | 1.0 |
| 0,01 | 1.273170731707317 |
| 0,02 | 1.420054200542005 |
| 0,1 | 1.665582655826558 |
| 0,5 | 1.792953929539295 |
| met | 2.123035230352303 |***
***
**
Folic Acid (mM)
**
***
**
***
Growth Fold Change
Growth Fold Change
MET
0,009
0,01
0,02
0,1
0,5
mM
0,009
0,01
0,02
0,1
0,5
mM
