# Supplementary material for: Folate can promote the methionine-dependent reprogramming of glioblastoma cells towards pluripotency
Source: Cell Death Dis. 2019 Aug 8;10(8):596. doi: 10.1038/s41419-019-1836-2 (PMC6687714; doi:10.1038/s41419-019-1836-2)
Supplement: Supplementary file 6 — Supplemental Figure SI5 [file 41419_2019_1836_MOESM6_ESM.pptx]

## Slide 1
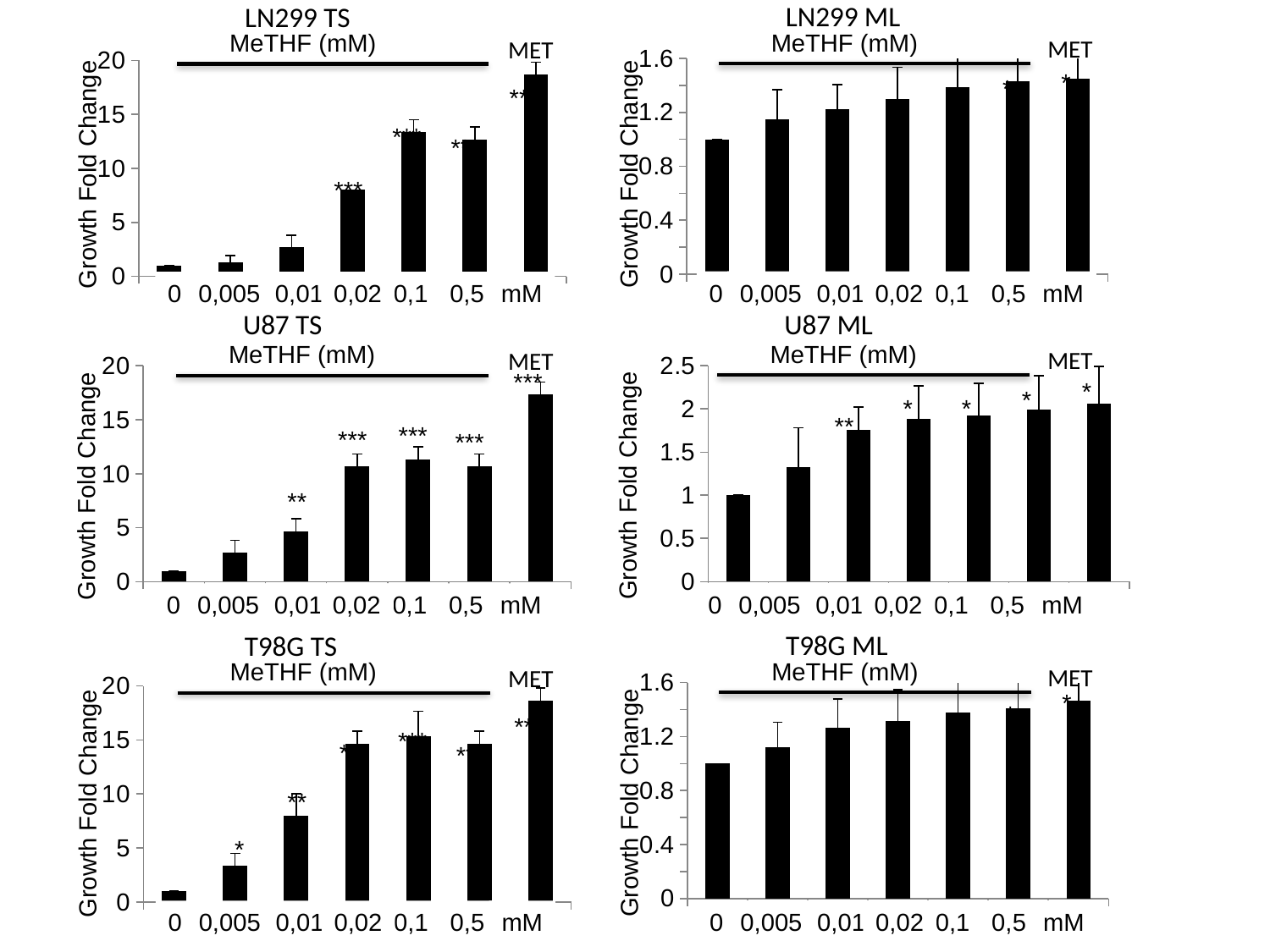

LN299 ML
LN299 TS
MeTHF (mM)
MeTHF (mM)
MET
MET
### Chart
| Category | fold av |
|---|---|
| 0 | 1.0 |
| 0.005 | 1.150877192982456 |
| 0.01 | 1.221637426900585 |
| 0.02 | 1.300730994152047 |
| 0.1 | 1.385818713450292 |
| 0.5 | 1.427923976608187 |
| met | 1.450146198830409 |
### Chart
| Category | moyenne fc |
|---|---|
| 0 | 1.0 |
| 0.005 | 1.333333333333333 |
| 0.01 | 2.666666666666666 |
| 0.02 | 8.0 |
| 0.1 | 13.33333333333333 |
| 0.5 | 12.66666666666667 |
| met | 18.66666666666667 |*
*
***
***
***
Growth Fold Change
Growth Fold Change
***
0
0,005
0,01
0,02
0,1
0,5
mM
0
0,005
0,01
0,02
0,1
0,5
mM
U87 ML
U87 TS
MeTHF (mM)
MeTHF (mM)
MET
MET
### Chart
| Category | moyenne fc |
|---|---|
| 0 | 1.0 |
| 0.005 | 2.666666666666666 |
| 0.01 | 4.666666666666667 |
| 0.02 | 10.66666666666667 |
| 0.1 | 11.33333333333333 |
| 0.5 | 10.66666666666667 |
| met | 17.33333333333328 |
### Chart
| Category | fold av |
|---|---|
| 0 | 1.0 |
| 0.005 | 1.322222222222222 |
| 0.01 | 1.752777777777778 |
| 0.02 | 1.88888888888889 |
| 0.1 | 1.927777777777778 |
| 0.5 | 1.997222222222222 |
| met | 2.058333333333333 |***
*
*
*
*
**
***
***
***
Growth Fold Change
Growth Fold Change
**
0
0,005
0,01
0,02
0,1
0,5
mM
0
0,005
0,01
0,02
0,1
0,5
mM
T98G ML
T98G TS
MeTHF (mM)
MeTHF (mM)
MET
MET
### Chart
| Category | fold av |
|---|---|
| 0 | 1.0 |
| 0.005 | 1.119241192411924 |
| 0.01 | 1.263685636856368 |
| 0.02 | 1.316260162601626 |
| 0.1 | 1.376964769647696 |
| 0.5 | 1.407317073170732 |
| met | 1.468021680216802 |
### Chart
| Category | moyenne fc |
|---|---|
| 0 | 1.0 |
| 0.005 | 3.333333333333333 |
| 0.01 | 8.0 |
| 0.02 | 14.66666666666667 |
| 0.1 | 15.33333333333333 |
| 0.5 | 14.66666666666667 |
| met | 18.66666666666667 |*
*
***
***
***
***
**
Growth Fold Change
Growth Fold Change
*
0
0,005
0,01
0,02
0,1
0,5
mM
0
0,005
0,01
0,02
0,1
0,5
mM
