# Supplementary material for: Folate can promote the methionine-dependent reprogramming of glioblastoma cells towards pluripotency
Source: Cell Death Dis. 2019 Aug 8;10(8):596. doi: 10.1038/s41419-019-1836-2 (PMC6687714; doi:10.1038/s41419-019-1836-2)
Supplement: Supplementary file 7 — Supplemental Figure SI6 [file 41419_2019_1836_MOESM7_ESM.pptx]

## Slide 1
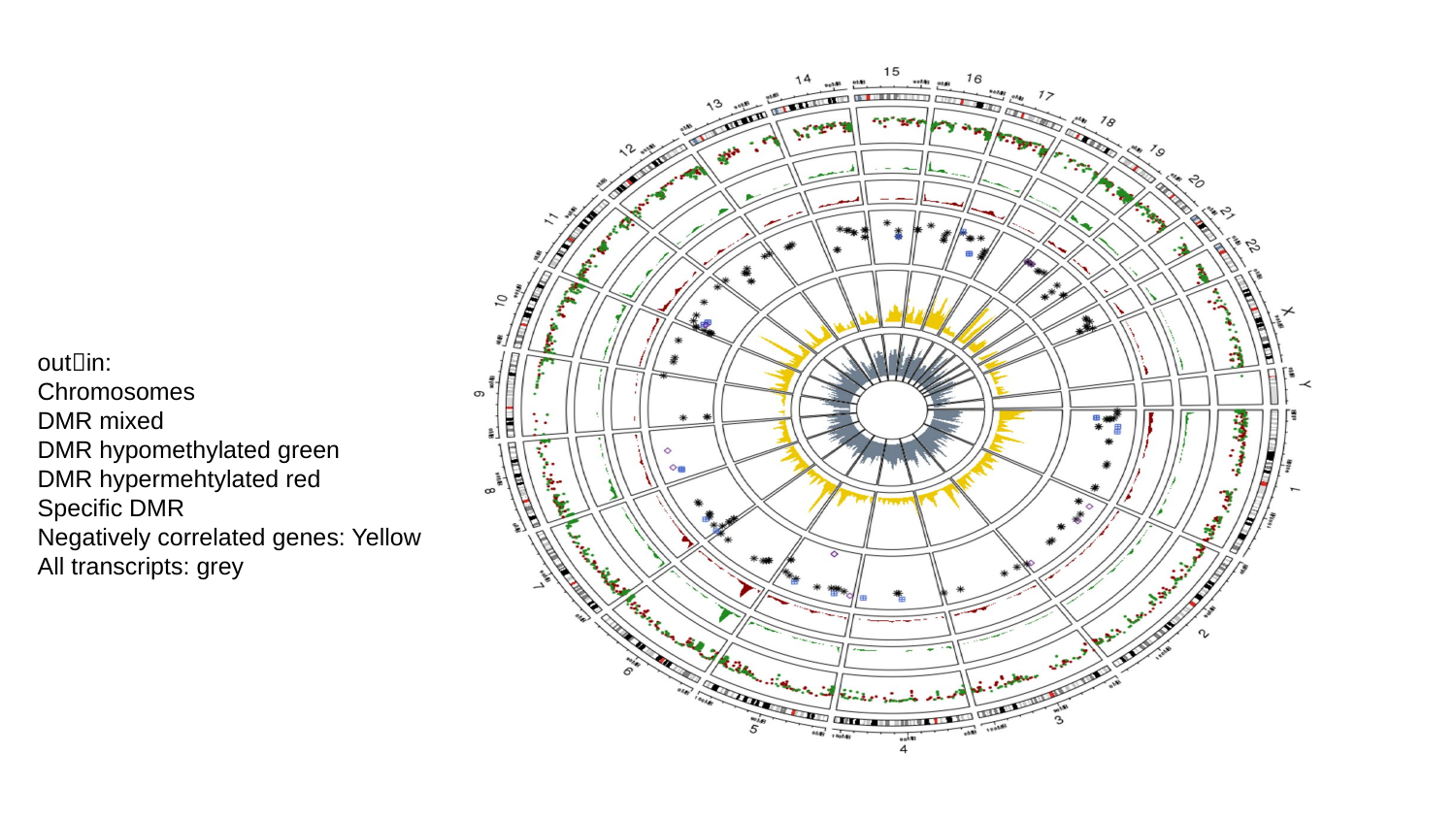

outin:
Chromosomes
DMR mixed
DMR hypomethylated green
DMR hypermehtylated red
Specific DMR
Negatively correlated genes: Yellow
All transcripts: grey
