# Supplementary material for: Folate can promote the methionine-dependent reprogramming of glioblastoma cells towards pluripotency
Source: Cell Death Dis. 2019 Aug 8;10(8):596. doi: 10.1038/s41419-019-1836-2 (PMC6687714; doi:10.1038/s41419-019-1836-2)
Supplement: Supplementary file 8 — Supplemental Figure SI7 [file 41419_2019_1836_MOESM8_ESM.pptx]

## Slide 1
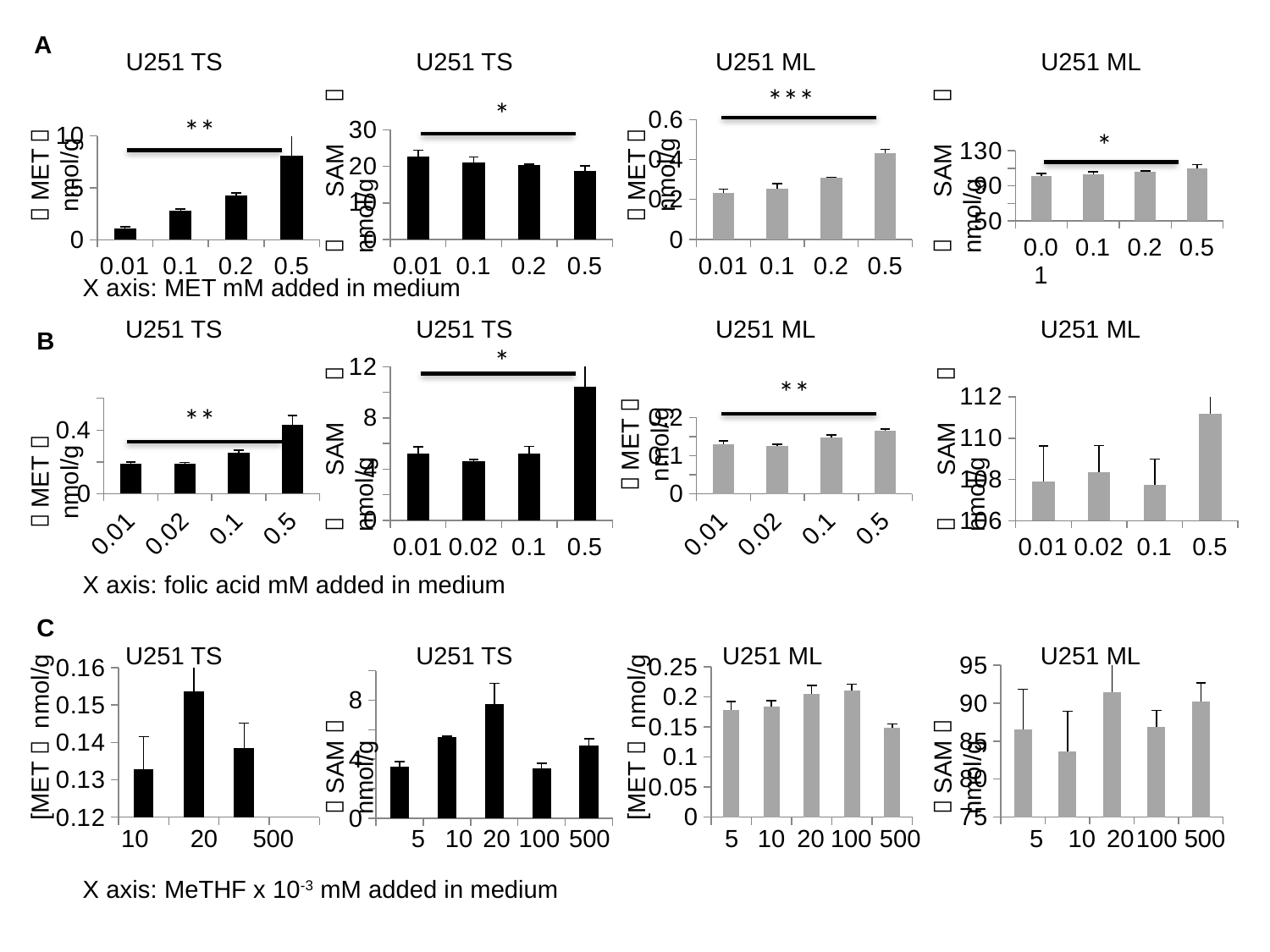

A
U251 TS
U251 TS
U251 ML
U251 ML
***
*
### Chart
| Category | |
|---|---|
| 0.01 | 0.230648435581936 |
| 0.1 | 0.253809496255068 |
| 0.2 | 0.307915630727377 |
| 0.5 | 0.430465019458989 |**
### Chart
| Category | |
|---|---|
| 0.01 | 22.73176706297044 |
| 0.1 | 21.09929516934595 |
| 0.2 | 20.30645472709924 |
| 0.5 | 18.69707711046845 |
### Chart
| Category | |
|---|---|
| 0.01 | 1.094329673223463 |
| 0.1 | 2.754480505775355 |
| 0.2 | 4.288484862385305 |
| 0.5 | 8.082742321098564 |*
### Chart
| Category | |
|---|---|
| 0.01 | 101.3385575802361 |
| 0.1 | 103.5656504424766 |
| 0.2 | 106.4936972855408 |
| 0.5 | 109.7876652766976 |［SAM］ nmol/g
［SAM］ nmol/g
［MET］ nmol/g
［MET］ nmol/g
X axis: MET mM added in medium
U251 TS
U251 TS
U251 ML
U251 ML
B
*
### Chart
| Category | |
|---|---|
| 0.01 | 5.193796223367982 |
| 0.02 | 4.605026256804786 |
| 0.1 | 5.23180312382763 |
| 0.5 | 10.4609977064754 |**
### Chart
| Category | |
|---|---|
| 0.01 | 107.8921717147105 |
| 0.02 | 108.3590278689577 |
| 0.1 | 107.7486730870886 |
| 0.5 | 111.1867746579027 |**
### Chart
| Category | |
|---|---|
| 0.01 | 0.186380272624643 |
| 0.02 | 0.190741112531056 |
| 0.1 | 0.258713978205158 |
| 0.5 | 0.43230561832334 |
### Chart
| Category | |
|---|---|
| 0.01 | 0.130533968106102 |
| 0.02 | 0.125911715347546 |
| 0.1 | 0.147724579226435 |
| 0.5 | 0.164990992450218 |［MET］ nmol/g
［SAM］ nmol/g
［SAM］ nmol/g
［MET］ nmol/g
X axis: folic acid mM added in medium
C
U251 TS
U251 TS
U251 ML
U251 ML
### Chart
| Category | |
|---|---|
| 0.005 | 86.5082869903009 |
| 0.01 | 83.61623492355098 |
| 0.02 | 91.42058071140109 |
| 0.1 | 86.8312530609211 |
| 0.5 | 90.1605608425511 |
### Chart
| Category | |
|---|---|
| 0.005 | 0.178080667978642 |
| 0.01 | 0.184139507708814 |
| 0.02 | 0.205210075366601 |
| 0.1 | 0.21001757585825 |
| 0.5 | 0.149082642884118 |
[unsupported chart]
### Chart
| Category | |
|---|---|
| 0.005 | 3.517644614194486 |
| 0.01 | 5.518645532757898 |
| 0.02 | 7.723453910667247 |
| 0.1 | 3.358536682469178 |
| 0.5 | 4.94844825742861 |［SAM］ nmol/g
［SAM］ nmol/g
[MET］ nmol/g
[MET］ nmol/g
10
20
500
5
10
20
100
500
5
10
20
100
500
5
10
20
100
500
X axis: MeTHF x 10-3 mM added in medium
