# Supplementary material for: Folate can promote the methionine-dependent reprogramming of glioblastoma cells towards pluripotency
Source: Cell Death Dis. 2019 Aug 8;10(8):596. doi: 10.1038/s41419-019-1836-2 (PMC6687714; doi:10.1038/s41419-019-1836-2)
Supplement: Supplementary file 9 — Supplemental Figure SI8 [file 41419_2019_1836_MOESM9_ESM.pptx]

## Slide 1
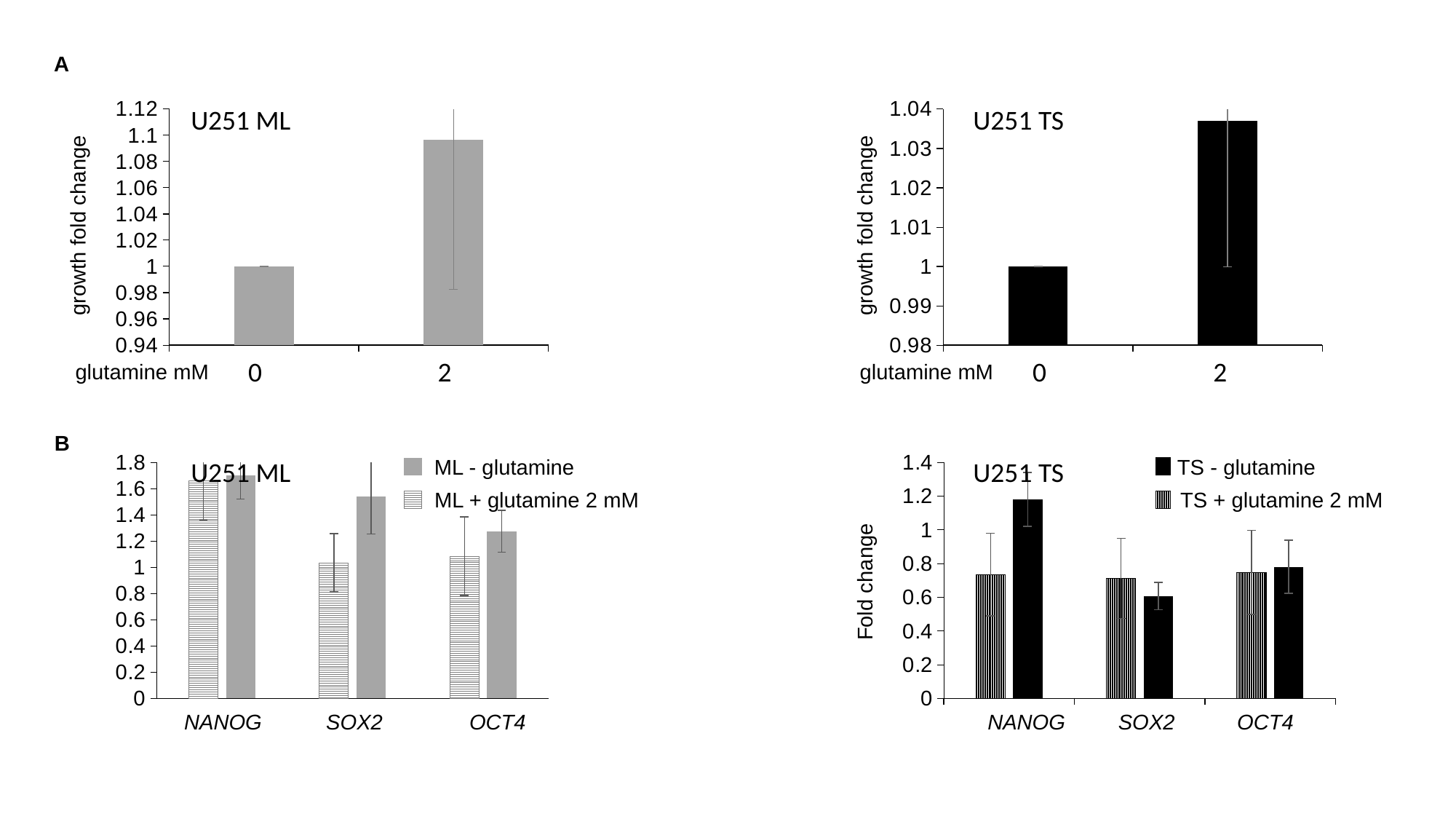

A
### Chart
| Category | |
|---|---|
| 0mM | 1.0 |
| 2mM | 1.096593478980169 |
### Chart
| Category | |
|---|---|
| 0mM | 1.0 |
| 2mM | 1.037037037037037 |U251 ML
U251 TS
growth fold change
growth fold change
0
2
0
2
glutamine mM
glutamine mM
B
### Chart
| Category | ADH+ glutamine | ADH- glutamine |
|---|---|---|
| nanog | 1.657915035284265 | 1.701709722830064 |
| sox2 | 1.034759191653103 | 1.538940673728186 |
| oct | 1.084521303502244 | 1.275109552800656 |
### Chart
| Category | TS+ glutamine | TS- glutamine |
|---|---|---|
| nanog | 0.734719509827243 | 1.181440752477257 |
| sox2 | 0.712971965146306 | 0.607704060533079 |
| oct | 0.748388267267602 | 0.78131849542561 |ML - glutamine
TS - glutamine
U251 ML
U251 TS
ML + glutamine 2 mM
TS + glutamine 2 mM
Fold change
NANOG
SOX2
OCT4
NANOG
SOX2
OCT4
