# Supplementary material for: Folate can promote the methionine-dependent reprogramming of glioblastoma cells towards pluripotency
Source: Cell Death Dis. 2019 Aug 8;10(8):596. doi: 10.1038/s41419-019-1836-2 (PMC6687714; doi:10.1038/s41419-019-1836-2)
Supplement: Supplementary file 11 — Supplemental Table SI2 [file 41419_2019_1836_MOESM11_ESM.pptx]

## Slide 1
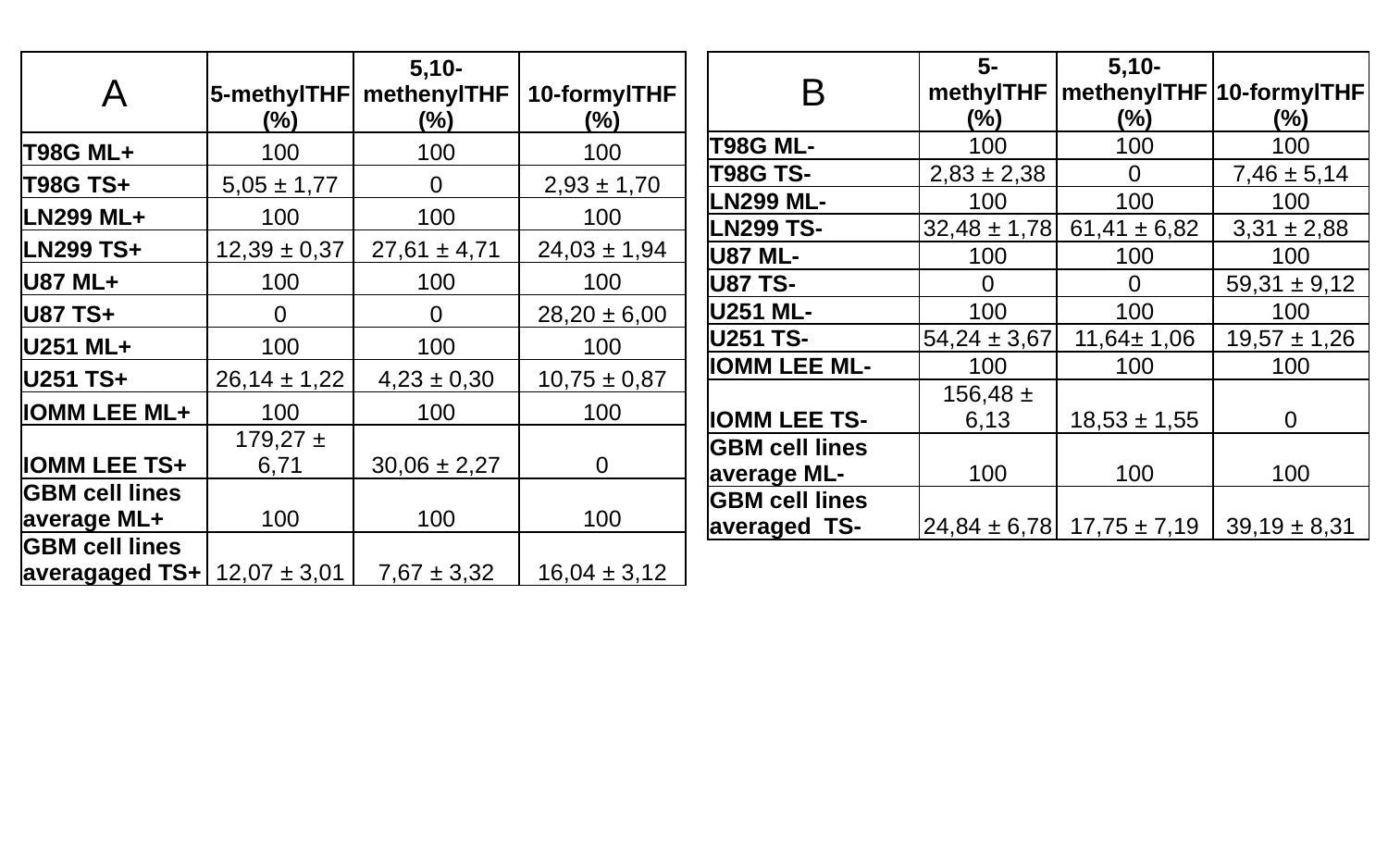

| A | 5-methylTHF (%) | 5,10-methenylTHF (%) | 10-formylTHF (%) |
| --- | --- | --- | --- |
| T98G ML+ | 100 | 100 | 100 |
| T98G TS+ | 5,05 ± 1,77 | 0 | 2,93 ± 1,70 |
| LN299 ML+ | 100 | 100 | 100 |
| LN299 TS+ | 12,39 ± 0,37 | 27,61 ± 4,71 | 24,03 ± 1,94 |
| U87 ML+ | 100 | 100 | 100 |
| U87 TS+ | 0 | 0 | 28,20 ± 6,00 |
| U251 ML+ | 100 | 100 | 100 |
| U251 TS+ | 26,14 ± 1,22 | 4,23 ± 0,30 | 10,75 ± 0,87 |
| IOMM LEE ML+ | 100 | 100 | 100 |
| IOMM LEE TS+ | 179,27 ± 6,71 | 30,06 ± 2,27 | 0 |
| GBM cell lines average ML+ | 100 | 100 | 100 |
| GBM cell lines averagaged TS+ | 12,07 ± 3,01 | 7,67 ± 3,32 | 16,04 ± 3,12 |
| B | 5-methylTHF (%) | 5,10-methenylTHF (%) | 10-formylTHF (%) |
| --- | --- | --- | --- |
| T98G ML- | 100 | 100 | 100 |
| T98G TS- | 2,83 ± 2,38 | 0 | 7,46 ± 5,14 |
| LN299 ML- | 100 | 100 | 100 |
| LN299 TS- | 32,48 ± 1,78 | 61,41 ± 6,82 | 3,31 ± 2,88 |
| U87 ML- | 100 | 100 | 100 |
| U87 TS- | 0 | 0 | 59,31 ± 9,12 |
| U251 ML- | 100 | 100 | 100 |
| U251 TS- | 54,24 ± 3,67 | 11,64± 1,06 | 19,57 ± 1,26 |
| IOMM LEE ML- | 100 | 100 | 100 |
| IOMM LEE TS- | 156,48 ± 6,13 | 18,53 ± 1,55 | 0 |
| GBM cell lines average ML- | 100 | 100 | 100 |
| GBM cell lines averaged TS- | 24,84 ± 6,78 | 17,75 ± 7,19 | 39,19 ± 8,31 |
